# A Core Effector MoPce1 Is Required for the Pathogenicity of *Magnaporthe oryzae* by Modulating Catalase‐Mediated H_2_O_2_ Homeostasis in Rice

**DOI:** 10.1111/mpp.70206

**Published:** 2026-01-16

**Authors:** Jianqiang Huang, Xiaomin Chen, Huimin Bai, Dao Zhou, Hongxia Zhang, Lifan Ke, Shuhui Lin, Xiuxiu Li, Zhenhui Zhong, Zonghua Wang, Huakun Zheng

**Affiliations:** ^1^ Fujian Universities Key Laboratory for Plant‐Microbe Interaction, College of Plant Protection Fujian Agriculture and Forestry University Fuzhou China; ^2^ State Key Laboratory of Agricultural and Forestry Biosecurity Fujian Agriculture and Forestry University Fuzhou China; ^3^ Ministry of Education Key Laboratory for Bio‐Resource and Eco‐Environment, College of Life Sciences, State Key Laboratory of Hydraulics and Mountain River Engineering Sichuan University Chengdu China; ^4^ Fuzhou Institute of Oceanography Minjiang University Fuzhou China

**Keywords:** core effector, OsCATC, reactive oxygen species, resistance, rice blast fungus

## Abstract

Plant pathogens employ a diverse array of effectors to facilitate host colonisation, including evolutionarily conserved core effectors. In this study, we identified MoPce1, a CAP/PR‐1 domain‐containing protein widely distributed among fungal species, as a key virulence factor in *Magnaporthe oryzae*. Among 72 putative core effectors (PCEs), MoPce1 was found to be essential for pathogenicity but dispensable for asexual development. It localises to biotrophic interfacial complex (BIC) in invasive hyphae (IHs) and to the cytoplasm in *Nicotiana* *benthamiana* leaves and rice protoplasts. Ectopic expression of a signal peptide‐deleted variant of *MoPCE1* (*MoPCE1* ^*Δsp*^) in rice compromised blast resistance and suppressed the reactive oxygen species (ROS) burst. Notably, MoPce1 lacks the conserved cysteine residues essential for sterol‐binding in the CAP domain, suggesting its potential association with a novel ligand. Further investigation revealed that MoPce1 interacts with rice catalase OsCATC, specifically via the C1 fragment (231–360 aa). Disruption of *OsCATC* (*oscatc*) enhanced rice blast resistance and triggered a stronger ROS burst. Collectively, our results indicate that MoPce1 targets OsCATC to disrupt ROS homeostasis and suppress host immunity, thereby facilitating infection.

## Graphical abstract

MoPce1, an atypical CAP/PR‐1 family effector, is essential for *Magnaporthe oryzae* virulence. MoPce1 functions by interacting with the rice catalase OsCATC to disrupt ROS homeostasis, thereby promoting host susceptibility.

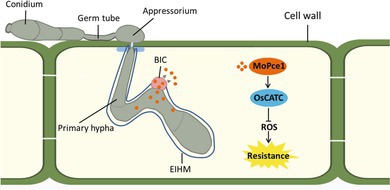

## 1 Introduction

Plants have evolved a two‐tiered immune defence system: pathogen‐associated molecular pattern (PAMP)‐triggered immunity (PTI) and effector‐triggered immunity (ETI) (Jones and Dangl 2006; Yuan et al. 2021). To counteract these defences, pathogens employ diverse strategies, including the secretion of a wide array of effectors with specialised functions (Giraldo and Valent 2013). While most known effectors are small, lineage‐specific secreted proteins lacking characterised functional domains, a subset—termed ‘core effectors’—are evolutionarily conserved and widely distributed across pathogen species (Morgan and Kamoun 2007; Irieda et al. 2019; Hoang et al. 2021; Chen, King, et al. 2023).

The CAP (cysteine‐rich secretory protein (CRISP)/antigen 5/pathogenesis related‐1) superfamily proteins are widely distributed across prokaryotes and eukaryotes (Abraham and Chandler 2017). These proteins play critical roles in diverse biological processes, including development and immune regulation in animals and plants (Gibbs et al. 2008; Han et al. 2023), as well as virulence in pathogens (Zhao et al. 2023). In fungal pathogens, CAP proteins are essential for virulence. For instance, in *Candida albicans* , deletion of *RBE1* or *RBT4* caused significant attenuation in pathogenicity (Röhm et al. 2013). In *Fusarium oxysporum* and *Fusarium graminearum*, the PR‐1‐like proteins are required for virulence on mammalian and plant hosts (Prados‐Rosales et al. 2012; Lu and Edwards 2018). In *Fusarium verticillioides*, *FvSCP1* is required for virulence on maize (Zhang et al. 2018). In the wheat stripe rust fungus *Puccinia striiformis* f. sp. *tritici*, three PsCAP proteins contribute to pathogenicity (Zhao et al. 2023). In *Cytospora chrysosperma* , CcCAP1 suppresses poplar immunity (Han et al. 2021). The CAP domain and the CAPE (CAP‐derived peptides) motif are implicated in the plant–microbe interactions (Lozano‐Torres et al. 2012, 2014; Luo et al. 2023). Although fungal CAP proteins have been linked to lipid export and sterol binding in fungi (Choudhary and Schneiter 2012), their functional mechanisms remain largely elusive. A recent breakthrough revealed that *Ustilago maydis* exploits maize cathepsin B‐like 3 (CatB3) to cleave the PR1‐like protein UmPR‐1La, generating functional CAPE‐like peptides that disrupt plant CAPE‐mediated immunity (Lin et al. 2023). Despite these advances, the functional repertoire of CAP proteins—particularly those lacking conserved cysteine residues (Schneiter and Di Pietro 2013) or the CAPE (PxGNxxxxxPY) motif (Lin et al. 2023)—remains poorly understood. Emerging evidence highlights diverse targeting mechanisms: potato PR1 proteins suppress *Phytophthora infestans* pathogenicity by targeting AMPK kinase complex subunits (Luo et al. 2023); the potato cyst nematode PR1‐like protein Gr‐VAP1 inhibits tomato immune responses by interacting with the papain‐like cysteine protease Rcr3^pim^ in plants lacking the *Cf‐2* resistance gene (Lozano‐Torres et al. 2014). These findings provide novel insights into the functional diversity and evolutionary adaptation of CAP proteins.

Catalase (CAT) is a ubiquitous antioxidant enzyme that catalyses the decomposition of H_2_O_2_ produced during various cellular processes, including photorespiration, mitochondrial electron transport and fatty acid β‐oxidation (Yang and Poovaiah 2002). Rice ( *Oryza sativa* ) possesses three *CAT* genes—*OsCATA*, *OsCATB* and *OsCATC*—that share 10 conserved domains (Hu et al. 2016) and exhibit functional conservation (Wang et al. 2016). Despite these similarities, their divergent expression patterns suggest distinct roles (Lin et al. 2011; Ye et al. 2011; Zhang et al. 2016; Liao et al. 2022). Notably, OsCATC plays a crucial role in blast resistance through multiple mechanisms, including modulating starch metabolism (Liao et al. 2022), and scavenging H_2_O_2_ via the AvrPiz‐t‐ROD1‐CATs‐APIP6/RIP1 immune regulatory network (You et al. 2022). Importantly, its H_2_O_2_‐scavenging activity requires phosphorylation by OsCPK12 (Wang et al. 2024). While these findings highlight the importance of OsCATC in rice immunity, whether it serves as a target for additional blast effector proteins remains an open question.

*Magnaporthe oryzae* (syn*. Pyricularia oryzae*) causes devastating annual yield losses sufficient to feed approximately 60 million people worldwide (Valent 2021; Zhang et al. 2024). Although several core effectors in *M. oryzae* have been functionally characterised—including Slp1 (Mentlak et al. 2012), NIS1 (Irieda et al. 2019), MC69 (Saitoh et al. 2012), BAS2 (Mosquera et al. 2009), and conserved domain‐containing effectors like the CFEM protein MoCDIP2 (Chen et al. 2013)—the majority remain uncharacterised. In this study, we identified 72 putative core effectors (PCEs) and demonstrate that MoPce1 (MGG_05100) is essential for full virulence in *M. oryzae* . Our mechanistic investigation revealed that MoPce1 likely suppresses plant immunity by modulating the H_2_O_2_‐scavenging activity of rice catalase OsCATC.

## 2 Results

### 2.1 Identification of Putative Core Effectors in Fungal Pathogen

We identified putative core effectors (PCEs) based on the following criteria: (1) Presence of a signal peptide but absence of transmembrane domains; and (2) conservation across all six tested *Pyricularia*/*Magnaporthe* isolates (70–15, BR32, DS0505, EI9604, SV9610 and P1609), as well as in *Colletotrichum gloeosporioides* and *Ustilago maydis* . A total of 72 PCEs was identified (Table S1), two of which had been previously reported (Saitoh et al. 2012). Functional analysis of seven PCEs revealed that *MoPCE1* (MGG_05100)—encoding a CAP‐domain protein (containing the cysteine‐rich secretory protein [CRISP]/antigen 5/pathogenesis‐related 1 [PR‐1] motif) (Han et al. 2021)—serves as a key virulence factor in *M. oryzae* (Figure S1; Table S2).

Multiple sequence alignment revealed that MoPce1 retains all conserved structural motifs characteristic of CAP proteins, including the four α‐helices (α1–4) and three β‐sheets (β1–3), although some residuals in these motifs show variation. Notably, the three conserved cysteine residues in β2 and β3 were substituted with other amino acids (Figure S2A). Phylogenetic analysis demonstrated that MoPce1 clusters most closely with homologues from other fungal species, showing high similarity to *M. oryzae* variants and moderate similarity to CcCap2 ( *C. chrysosperma* ), FGSG_03109 (*F. graminearum*), and FOXG_14109 (*F. oxysporum*). In contrast, it exhibited only a distant relationship to PR1 homologues from *Arabidopsis thaliana* and *Nicotiana tabacum* , and was most divergent from CAP proteins of other species, such as the yeast PRY proteins (Figure S2B).

### 2.2 MoPce1 Is Required for the Pathogenicity but Not Asexual Development in *M. oryzae*

**FIGURE 1:**
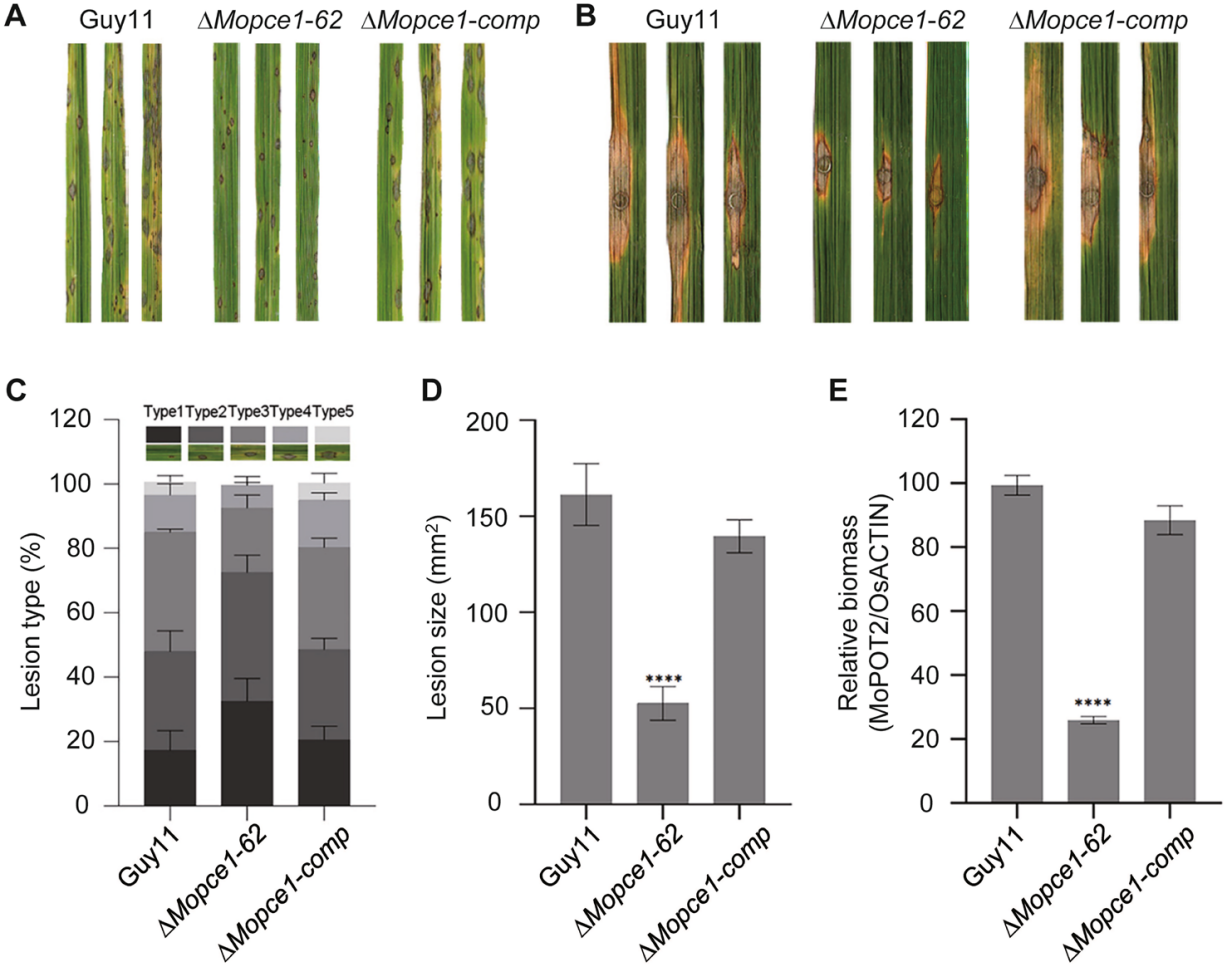
MoPce1 is required for pathogenicity in *Magnaporthe oryzae* . (A, B) Disease symptoms (A) and lesion types (B) on NPB rice seedlings following spray inoculation with Guy11 wild‐type (WT), Δ*Mopce1* mutant, and complemented strains. (C–E) Punch inoculation assays showing disease symptoms (C), lesion size (D) and relative fungal biomass (measured by quantitative PCR) (E) on NPB seedlings infected with WT, Δ*Mopce1*, and complemented strains. All experiments were performed with three biological replicates, yielding consistent results. Disease symptoms in (B) were photographed 10 days after inoculation. Statistical significance in (C) was analysed by two‐way ANOVA followed by Dunnett's multiple comparisons test (simple effects within columns), whereas panels (D) and (E) were analysed by one‐way ANOVA followed by Dunnett's multiple comparisons test, with Guy11 as the control. The data are shown as means ± standard error (*n* = 6). Statistical significance: ****p < 0.0001.

**FIGURE 2:**
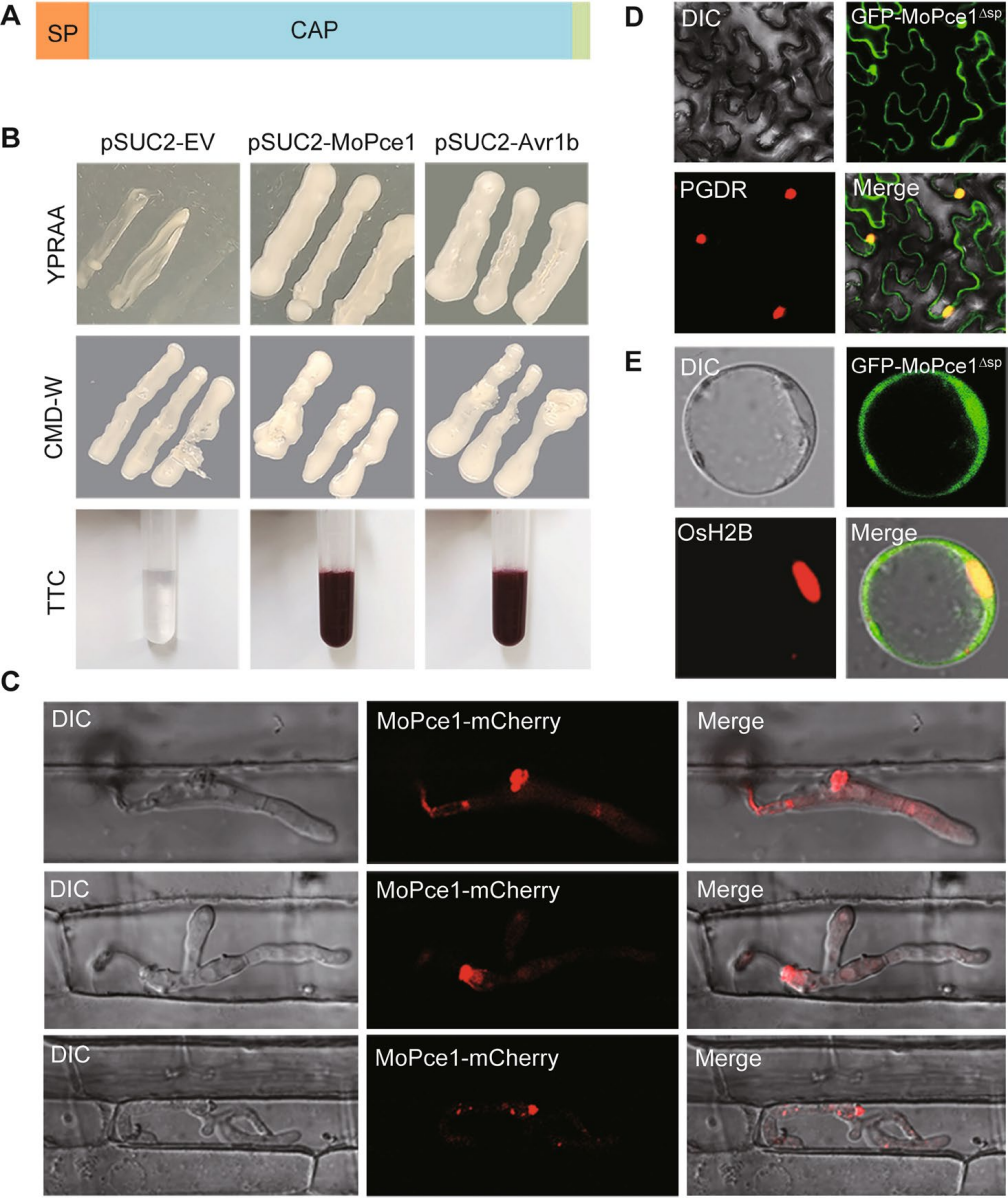
MoPce1 is a secreted protein. (A) MoPce1 contains an N‐terminal signal peptide (SP) and a CAP domain. (B) Functional validation of the MoPce1 signal peptide using the yeast signal peptide screen method. (C) MoPce1‐mCherry fluorescence accumulated in the biotrophic interfacial complex (BIC). (D, E) The GFP‐MoPce1 fusion localised to the nuclei of *Nicotiana* *benthamiana* leaf cells (D) and rice protoplasts (E) upon ectopic expression.

To further characterise the role of MoPce1 in asexual development and pathogenicity, we generated a ∆*MoPce1* mutant and its corresponding complementation strain, followed by phenotypic characterisation. The Δ*Mopce1* strain exhibited growth, conidiation and appressorium formation comparable to the wild type and complemented strains (Figure S3; Tables S3 and S4). However, pathogenicity assays revealed significant defects. In spray inoculation assays, the Δ*Mopce1* strain produced fewer lesions than the wild type and complemented strains (Figure 1A,B; Table S5). Similarly, in the punch inoculation assays, lesion size and fungal biomass (quantified by relative DNA content) were significantly reduced in the Δ*Mopce1* strain (Figure 2C–E). These results demonstrate that MoPce1 is required for full virulence but is dispensable for vegetative growth, conidiation or appressorial development in rice blast fungus.

### 2.3 MoPce1 is a Cytoplasmic Effector

To validate MoPce1 as a bona fide secreted protein, we first functionally assessed its predicted signal peptide (SP) using the yeast signal peptide screen method. The MoPce1_SP fusion enabled growth of *Saccharomyces cerevisiae* YTK12 strain on both CMD−W and YPRAA media, while also inducing the characteristic red colour reaction (Figure 2A,B). These results confirm that MoPce1 possesses a functional secretion signal.

To determine the subcellular localisation of MoPce1, we first verified the functionality of MoPce1‐mCherry and GFP‐MoPce1. Both fusion proteins successfully complemented the pathogenicity defects of the Δ*Mopce1* strain (Figure S4; Tables S6 and S7). Initial attempts to detect fluorescence using the native *MoPCE1* promoter were unsuccessful. We therefore employed the *PWL2* promoter to drive expression of *MoPCE1‐mCherry*. Live‐cell imaging revealed fluorescence accumulation in the biotrophic interfacial complex (BIC) during infection (Figure 2C), which suggests that MoPce1 is a cytoplasmic effector. To examine potential plant cell localisation, we ectopically expressed *MoPCE1‐GFP* in *Nicotiana* *benthamiana* leaves and in rice protoplasts. In both systems, fluorescence accumulated in nuclei (Figure 2D,E), indicative of a cytoplasmic effector.

### 2.4 Ectopic Expression of MoPce1 in Rice Plants Compromises Immunity Against *M. oryzae*

**FIGURE 3:**
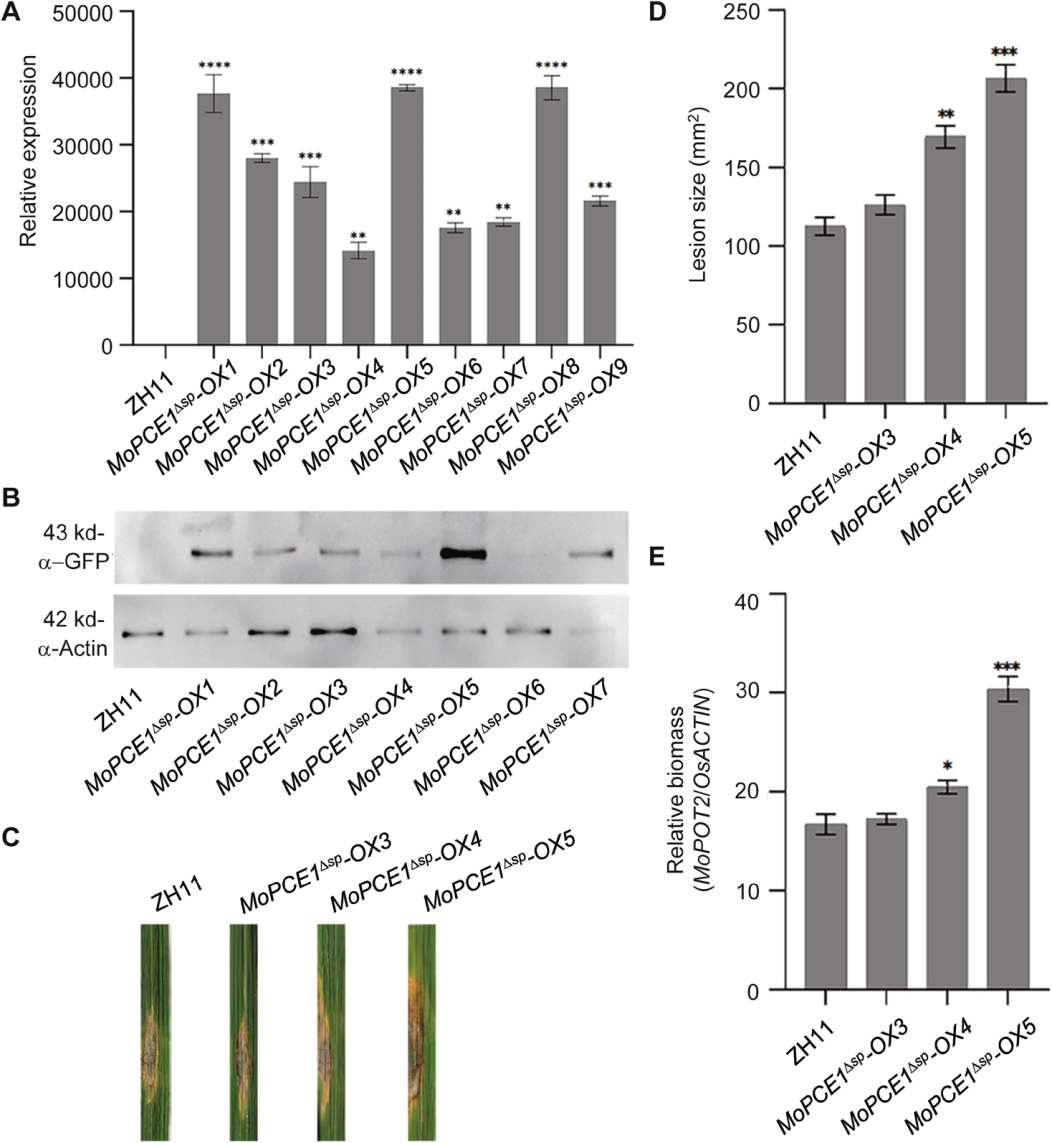
Ectopic expression of *MoPCE1* ^*ΔSP*^ in rice compromises resistance to *Magnaporthe oryzae* . (A,B) Reverse transcription‐quantitative PCR (A) and western blot (B) assays validated transgenic rice plants ectopically expressing *MoPCE1* ^*ΔSP*^ *‐GFP* fusion (*MoPCE1* ^*ΔSP*^ *‐OX*). (C–E) Ectopic expression of *MoPCE1* ^*ΔSP*^ *‐GFP* caused increased lesion size (C,D) and relative biomass (E) in transgenic rice plants compared with the ZH11 wild type. Disease symptoms were photographed 10 days after inoculation. Statistical analysis was performed using one‐way ANOVA followed by Dunnett's multiple comparisons test, with ZH11 as the control group. The data are shown as means ± standard error (*n* = 6). Three replications were performed with similar results. *p < 0.05, **p < 0.01, ***p < 0.001, ****p < 0.0001.

To further investigate the function of MoPce1 during the interaction between *M. oryzae* and rice, we generated transgenic rice lines that ectopically express the MoPCE1^ΔSP^‐GFP fusion. Nine individual lines expressing the *MoPCE1* ^*ΔSP*^ *‐GFP* fusion were identified using both reverse transcription‐quantitative PCR (RT‐qPCR) and western blot assays (Figure 3A,B; Table S8). We then chose three lines (#3, #4, and #5) representing different expression levels for further study. When challenged with the *M. oryzae* Guy11 spore solution in the punch inoculation assays, the size and relative biomass of lesions generated on MoPCE1^ΔSP^‐OX lines were larger than those of the wild‐type rice cultivar ZH11 (Figure 3C–E; Tables S9 and S10). These results suggest that transgenic rice plants ectopically expressing *MoPCE1* ^*ΔSP*^ are more susceptible to rice blast fungus, consistent with its role in the pathogenicity of *M. oryzae* .

**FIGURE 4:**
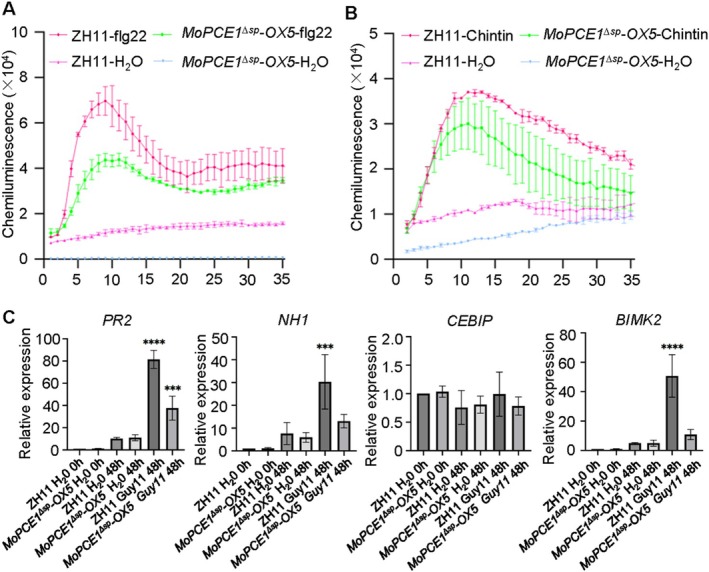
Ectopic expression of *MoPCE1* ^*ΔSP*^ in rice plants impairs their immunity. (A,B) Reactive oxygen species (ROS) burst in ZH11 wild type and the *MoPCE1* ^*ΔSP*^ *‐OX5* transgenic line treated with or without flg22 (A) or chitin (B). (C) Relative expression levels of the indicated pathogenesis‐related genes in ZH11 wild type and the *MoPCE1* ^*ΔSP*^ *‐OX5* transgenic line challenged with water or Guy11 spore solution. Statistical significance was assessed using two‐way ANOVA followed by Dunnett's multiple comparisons test (simple effects within rows), with ZH11–H_2_O as the control, for panels (A) and (B); one‐way ANOVA followed by Dunnett's multiple comparisons test, with H₂O 0 h ZH11 as the control, was used for panel (C). Values are presented as means ± standard error (*n* = 3). Three replications were performed with similar results. ***p < 0.001, ****p < 0.0001.

An investigation was conducted to assess the immune response of *MoPCE1* ^Δ*SP*^‐*OX* lines. Upon treatment with water, the reactive oxygen species (ROS) levels were lower in the *MoPCE1* ^*ΔSP*^ *‐OX* plants, which suggests that overexpression of *MoPCE1* reduces the accumulation of ROS (Figure 4A,B). Upon treatment with either flg22 or chitin, *MoPCE1* ^*ΔSP*^ *‐OX* plants exhibited a weaker burst of ROS compared to the wild‐type cultivar ZH11 (Figure 4A,B; Tables S11 and S12). We also found that following treatment with Guy11 spore solution, the related expression level of the pathogenesis‐related genes (PRs) in *MoPCE1* ^Δ*SP*^‐*OX* plants was lower than those in the ZH11 wild type (Figure 4C,D; Table S13). Taken together, these results suggest that the enhanced susceptibility of *MoPCE1* ^Δ*SP*^‐*OX* lines is attributed to compromised plant immunity.

### 2.5 Interaction Between MoPce1 and OsCATC

**FIGURE 5:**
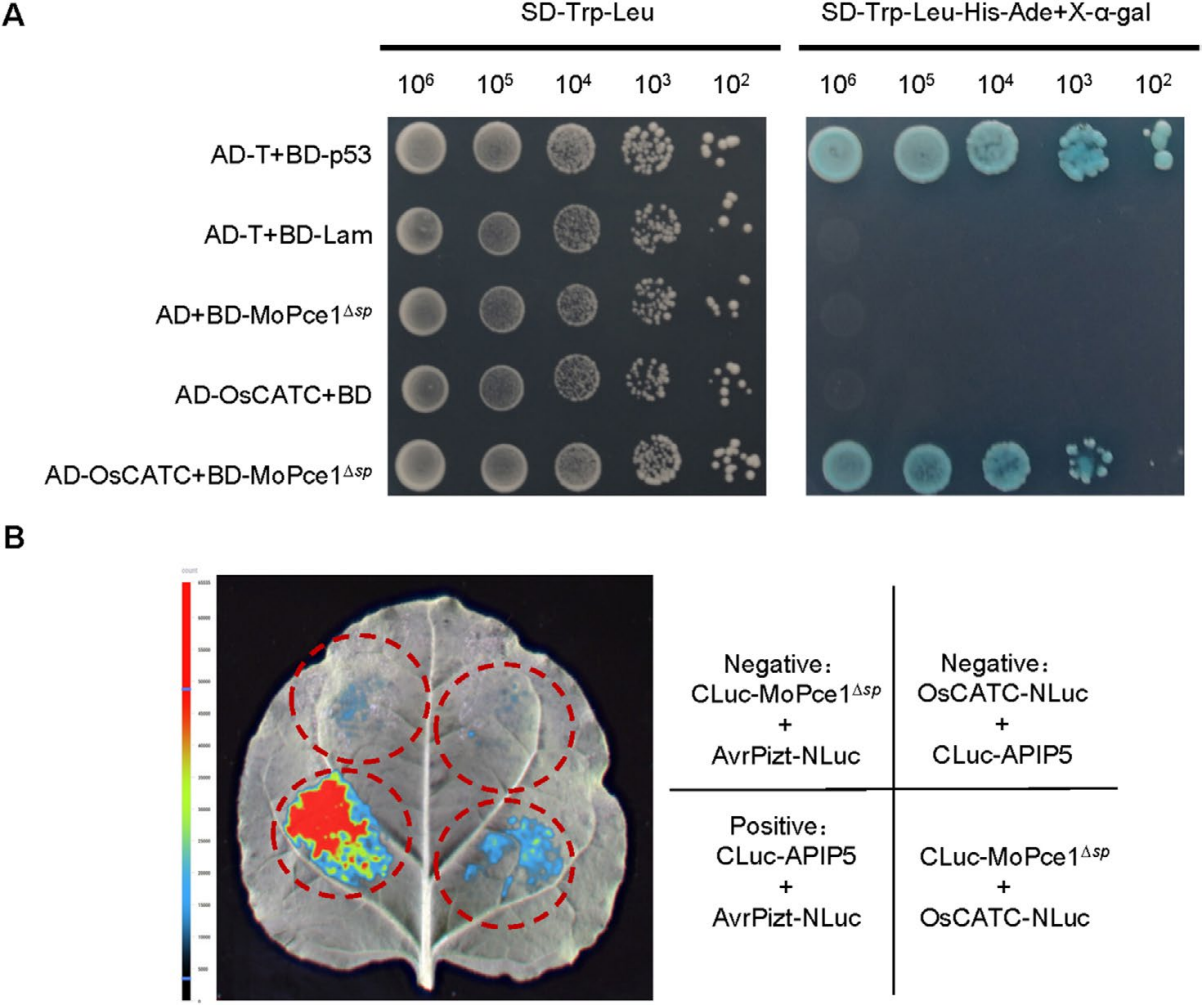
MoPce1 interacts with OsCATC. (A) Yeast‐two‐hybrid assays were performed to confirm the interaction between BD‐MoPce1^ΔSP^ and OsCATC‐AD. BD‐p53/AD‐T and BD‐Lam/AD‐T were used as positive and negative controls, respectively. (B) Luciferase complementation assay confirming the interaction between MoPce1 and OsCATC. The CLuc‐APIP5/AvrPizt‐NLuc pair was used as a positive control, while the CLuc‐MoPce1^Δsp^/AvrPizt‐NLuc and OsCATC‐NLuc/CLuc‐APIP5 pairs were used as negative controls.

To identify potential host targets of the *M. oryzae* CAP effector protein MoPce1 in rice, we performed a preliminary screening using co‐immunoprecipitation coupled with mass spectrometry (Co‐IP/MS) analysis using transgenic rice plants ectopically expressing *MoPCE1* ^*ΔSP*^ *‐GFP* (Table S14). Subsequent validation through yeast two‐hybrid (Y2H) assays and luciferase complementation assays (LCA) confirmed a specific interaction between MoPce1 and the catalase OsCATC (Figure 5). To further characterise the region of OsCATC required for this interaction, the protein was divided into three fragments (Figure S5A): the N fragment (1–230 amino acids [aa]), C1 fragment (231–360 aa), and C2 fragment (361–492 aa). Y2H assays revealed that the C1 fragment, rather than the N fragment or C2 fragment, is essential for the interaction between OsCATC and MoPce1 (Figure S5B).

### 2.6 OsCATC Negatively Regulates Rice Disease Resistance

**FIGURE 6:**
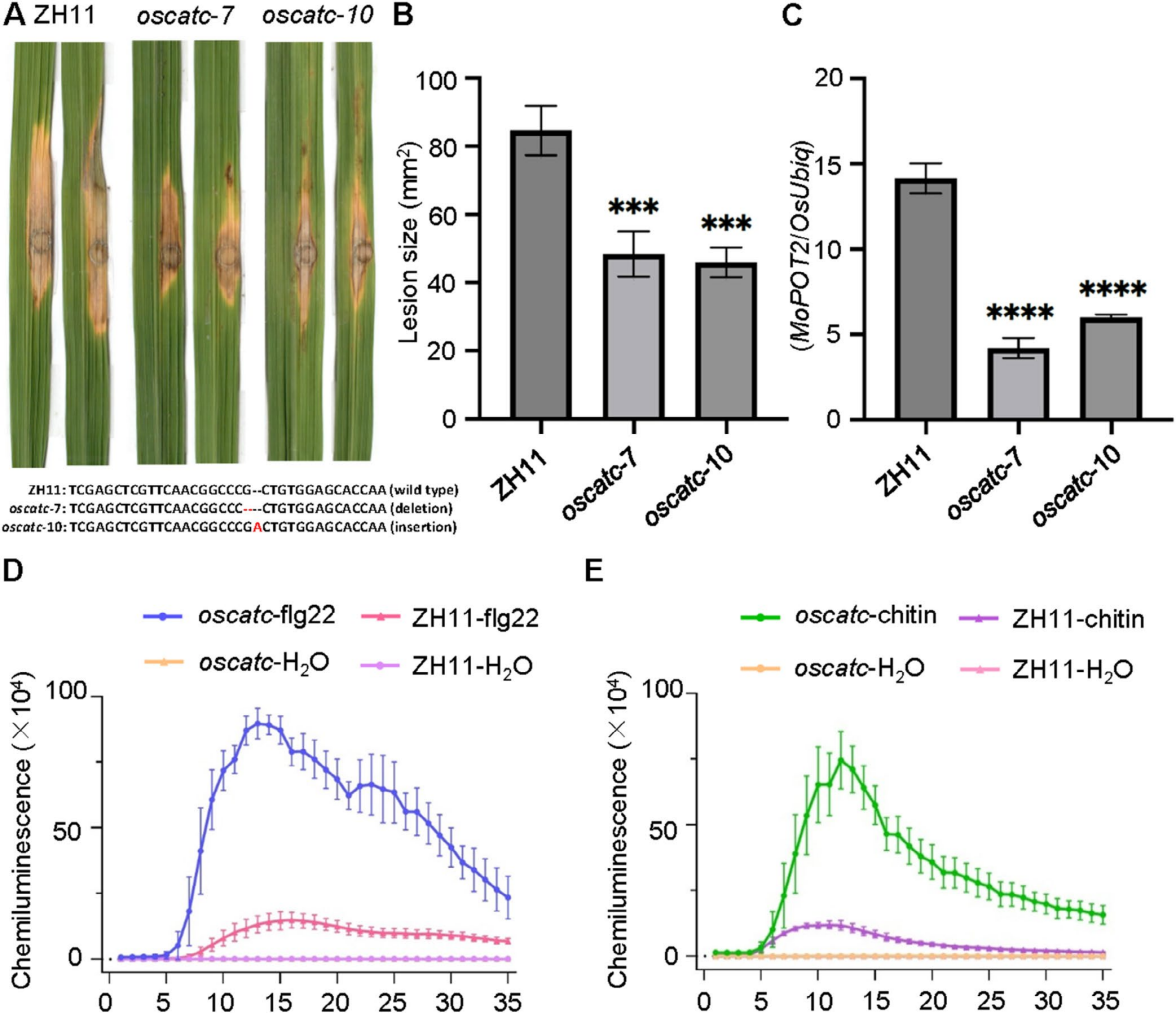
OsCATC negatively regulates rice immunity. (A) Punch inoculation assay. Spore solutions of *Magnaporthe oryzae* strain Guy11 were inoculated onto 6‐week‐old ZH11 and *oscatc* rice leaves. Disease symptoms were photographed at 10 days post‐inoculation (dpi). The data are shown as means ± standard error (*n* = 6). (B) Lesion area quantification. Lesion areas were measured using ImageJ software and statistically analysed. (C) Fungal biomass quantification. Genomic DNA was extracted from inoculated leaf tissues, and quantitative PCR was performed to determine the relative biomass of *M. oryzae* . (D) Reactive oxygen species (ROS) burst in ZH11 and *oscatc* rice leaves treated with flg22. (E) ROS burst in ZH11 and *oscatc* rice leaves treated with chitin. Statistical significance was assessed using one‐way ANOVA followed by Dunnett's multiple comparisons test, with H_2_O 0 h ZH11 as the control for panels (B) and (C); two‐way ANOVA followed by Dunnett's multiple comparisons test (simple effects within rows), with ZH11–Water as the control, was used for panels (D) and (E). Values are presented as means ± standard error (*n* = 3). Three replications were performed with similar results. ***p < 0.001, ****p < 0.0001.

To investigate the role of OsCATC in rice immunity, we generated an *OsCATC* knockout mutant (*oscatc*) in the ZH11 background. In punch inoculation assays, the lesion size on *oscatc* was significantly smaller than those on ZH11 (Figure 6A,B; Table S15). Quantitative PCR (qPCR) analysis further confirmed a significant reduction in fungal biomass at the infection sites of *oscatc* compared to ZH11 (Figure 6C; Table S16). These results indicate that OsCATC deletion enhances rice resistance against *M. oryzae* and suggest that OsCATC plays a negative regulatory role in host immunity.

To further validate this mechanism, rice leaves were treated with flg22 or chitin. Real‐time monitoring of the ROS burst via a microplate reader revealed that both elicitors triggered ROS production in ZH11 and *oscatc*. However, *oscatc* displayed significantly higher ROS accumulation compared to ZH11 (Figure 6D,E; Tables [Link], [Link]). These findings demonstrate that OsCATC suppresses the ROS burst during PTI, thus negatively regulating rice basal resistance against *M. oryzae* . The observed phenotypes align with previously reported roles of catalases in plant–pathogen interactions (You et al. 2022).

## 3 Discussion

In this study, we characterised the function of MoPCE1, a CAP domain‐containing core effector protein required for full virulence in *M. oryzae* . Furthermore, we found that MoPce1 targets the rice catalase OsCATC, thereby contributing to the compromised immune response in *MoPCE1*‐overexpressing transgenic rice.

The CAP domain‐containing proteins are broadly distributed across diverse eukaryotic species. They are characterised by two motifs that mediate sterol binding and fatty acid binding, and contain several conserved cysteine residues (Schneiter and Di Pietro 2013). In this study, we showed that MoPce1 contains all the conserved motifs but has lost all three conserved cysteine residues of the CAP domain (Figure S2A). Intriguingly, MoPce1 is phylogenetically closer to *Arabidopsis* PR1 than to orthologs from many fungal species, such as PRY1 from yeast (Figure S2B). This result is in agreement with the hypothesis that these CAP proteins could be expended and evolved independently in *M. oryzae* after the horizontal transfer from plants to fungi (Schneiter and Di Pietro 2013).

CAP domain‐containing proteins exhibit diversified functions across different fungal species. In the nonpathogenic yeast, PRY1 and PRY2 are required for the detoxification of plant‐derived compounds such as eugenol (Bakkali et al. 2008), while the cell wall protein Pry3 plays a negative role in mating (Cottier et al. 2020). In fungal pathogens, increasing evidence has demonstrated the involvement of CAP proteins in host–pathogen interactions. For example, RBT4 from *C. albicans* (Braun et al. 2000; Röhm et al. 2013), MpPR‐1 from *Moniliophthora perniciosa* (Teixeira et al. 2012), Fpr1 from *F. oxysporum* (Prados‐Rosales et al. 2012), FgPR‐1 L‐4 from *F. graminearum* (Lu and Edwards 2018), CAP proteins from the wheat stripe rust fungus *P. striiformis* f. sp. *tritici* (Zhao et al. 2023), CcCAP1 from *C. chrysosperma* (Han et al. 2021), and UmPR‐1La from *U. maydis* (Cottier et al. 2020) are all essential for the full virulence of these pathogens. In this study, we found that deletion of *MoPCE1* compromised the pathogenicity of the rice blast fungus (Figure 1). Additionally, ectopic expression of *MoPCE1* in rice attenuated the plant immune response (Figure 4), resulting in significantly increased susceptibility (Figure 3C–E). However, the Δ*Mopce1* strain still caused susceptible lesions. It is possible that other CAP proteins in *M. oryzae* exhibit functional redundancy during infection.

The CAP proteins may mediate fungal pathogen infection via diverse mechanisms. The participation of these proteins in sterol binding and lipid export—processes that are thought to contribute to pathogenicity—is well documented (Prados‐Rosales et al. 2012; Teixeira et al. 2012; Röhm et al. 2013; Darwiche et al. 2017; Lu and Edwards 2018). While direct evidence for the role of CAP domain‐mediated sterol binding during fungal pathogenesis is still lacking, a study of tobacco PR‐1 demonstrated that plants can inhibit pathogen growth by sequestering sterol from pathogens via the PR‐1 protein (Gamir et al. 2017). In *U. maydis* , UmPR‐1La not only mediates phenolic sensing and hyphal‐like formation but also releases CAPE‐like peptides to subvert plant immunity (Lin et al. 2023). Pathogens have evolved specific clades of CAP proteins that lack the conserved cysteine residues required for intramolecular disulphide bridges, and these proteins have been proposed to bind novel ligands (Schneiter and Di Pietro 2013). This functional diversity is further exemplified by other CAP family members, such as potato PR1 targeting subunits of the AMPK complex in *P. infestans* (Luo et al. 2023), and the venom allergen‐like effector protein Gr‐VAP1 from *Globodera rostochiensis* binding Rcr3pim (Lozano‐Torres et al. 2012). In line with these findings, the absence of the three cysteine residues in the MoPce1 CAP domain (Figure S2A) suggests that MoPce1 may also bind a non‐sterol ligand. We showed that MoPce1 localises to the BIC during infection (Figure 2C), indicative of its function as a cytoplasmic effector. This is consistent with its cytoplasmic localisation when ectopically expressed in *N. benthamiana* leaves and rice protoplasts (Figure 2D,E). Moreover, we found that MoPce1 interacted with OsCATC (Figure 5), a known negative regulator of rice immunity (You et al. 2022). It should be noted, however, that BIC localisation was only observed when MoPCE1 was expressed under the control of the *PWL2* promoter, not its native promoter. Given the influence of promoters on effector localisation (Khang et al. 2013), the BIC localisation of MoPce1 requires further validation.

Consistent with previous findings in the NPB background (You et al. 2022), our analysis revealed that *OsCATC* knockout plants in the ZH11 background also exhibited enhanced disease resistance (Figure 6A–C). Because OsCATC deficiency leads to excessive ROS accumulation during immune responses (Figure 6D,E), we propose that MoPce1 binding could enhance the enzymatic activity of OsCATC to promote ROS scavenging, thereby facilitating *M. oryzae* infection. However, the precise regulatory mechanism by which MoPce1 regulates OsCATC remains unclear. Structural analysis suggests that MoPce1 interacts with the C1 fragment (231–360 aa) of OsCATC (Figure S5)—a region distinct from the N‐terminal phosphorylation site (Ser11) targeted by OsCPK12 (Wang et al. 2024). This implies that MoPce1 is unlikely to modulate OsCPK12‐mediated phosphorylation of OsCATC. Interestingly, OsCATC is known to interact with APIP6, which suppresses H_2_O_2_ clearance by promoting OsCATC degradation (You et al. 2022). We hypothesise that MoPce1 may competitively inhibit APIP6‐mediated ubiquitination of OsCATC via steric hindrance; this would thereby stabilise OsCATC and sustain its ROS‐scavenging activity to promote pathogen virulence. The lower ROS level in the *MoPCE1* ^*ΔSP*^ *‐OX* lines (Figure 4A,B) may partially support this hypothesis. However, further experimental validation is required to fully elucidate the role of MoPce1 in manipulating host immunity.

In conclusion, we identified a putative core effector MoPCE1, which is a CAP/PR domain‐containing protein that is conserved across different species. The MoPce1 protein can be secreted and accumulated at BICs in the invasive hyphae, and is thought to be translocated into rice cells, where it interacts with the catalase protein OsCATC to regulate the ROS homeostasis. The results suggest that MoPce1 is required for the full virulence of *M. oryzae* by modulating OsCATC‐mediated ROS homeostasis.

## 4 Experimental Procedures

### 4.1 Identification of the PCE Proteins

To identify the PCEs, we searched for the homologues of the secreted proteins identified in the *Pyricularia penniseti* isolate P1609 (Zheng et al. 2018) using Orthofinder (Emms and Kelly 2015)—these homologues were retrieved from six *Pyricularia*/*Magnaporthe* isolates 70–15, BR32, DS0505, EI9604, SV9610 and P1609 (Zhong et al. 2016), as well as two additional species: *C. gloeosporioides* and *U. maydis* . A total of 72 proteins were identified as the PCE candidates (Table S1). After initial screening via pathogenicity analysis using the knockout mutants of seven genes (Table S2), MGG_05100, which encodes a protein with a CAP domain, was chosen for further investigation. The NCBI database and UniProt (Consortium 2015) were used to predict homologous proteins (both previously studied ones and CAP superfamily members) from selected species. SignalP v. 4.1 was used to predict signal peptides (https://services.healthtech.dtu.dk/services/SignalP‐4.1/). Additionally, we used MEGA X and ClustalX to construct a phylogenetic tree of CAP protein homologues. A multiple sequence alignment was performed using ClustalW (https://www.genome.jp/tools‐bin/clustalw) and ESPript (https://espript.ibcp.fr/ESPript/ESPript/index.php).

### 4.2 Culture Conditions of Fungal Strains

Manipulation of fungal strains was performed as described previously (Zhang et al. 2021). The indicated strains were cultured at 28°C in complete medium (CM: 6 g/L yeast extract, 6 g/L acid‐hydrolysed casein, 10 g/L sucrose, 20 g/L agarose). Fungal mycelia were harvested by culturing in liquid CM at 28°C in the dark. For the induction of conidiation, fungal blocks were transferred from CM plates to rice bran medium (RBM: 40 g/L rice bran, adjusted to pH 6.0–6.5 with NaOH, 20 g/L agar). The cultures were incubated in the dark at 28°C for 5–7 days, followed by 3 days of light exposure to obtain conidia.

### 4.3 Generation of Deletion Strains and Its Complemented Strains

To construct *MoPCE1* deletion mutants and complemented strains, we used the knockout vector pCX62 and the marker fusion method with polyethylene glycol (PEG)‐mediated transformation. The full‐length open reading frame (ORF) sequence of *MoPCE1* was deleted. First, the upstream and downstream flanking sequences of *MoPCE1* (973 bp and 1022 bp, respectively) were amplified using the primers MoPCE1‐AF/AR and MoPCE1‐BF/BR (Table S19). Then, the PCR products were inserted into the pCX62 vector containing the hygromycin phosphotransferase (*HPH*) cassette. Finally, the fusion fragment (AH and HB) was amplified and transformed into the protoplasts of Guy11. The transformed colonies were screened using the primer pairs MoPCE1tF/tR and MoPCE1UAF/H853 (Table S19). To confirm the deletion mutants, Southern blot analysis was performed as described previously (Liu et al. 2025). The genomic DNA isolated from the Guy11 wild type and the Δ*Mopce1* strains were digested with HindIII and hybridised with the A fragment of *MoPCE1*.

To construct complemented strains of *MoPCE1*, we amplified a 3.2 kb sequence containing the upstream and downstream region of *MoPCE1* using the primers 5100com_KpnIF/5100com_HindIIIR (Table S19), which was inserted into the pKNT vector. The resultant plasmid was then introduced into the Δ*Mopce1* protoplasts.

### 4.4 Pathogenicity and Live‐Cell Imaging Assays

Spore solution of each *M. oryzae* strain (5 × 10^4^ spores mL^−1^ in 0.02% Tween 20) was used to inoculate leaves of approximately 3‐week‐old rice plants. Disease symptoms caused by *M. oryzae* were recorded and the number of different types of lesions (type 1 to type 5) was calculated at 5–7 days after inoculation as described previously (Yang et al. 2024). Each strain was inoculated onto three pots of rice seedlings. Each pot had 6–8 seedlings. Approximately 10 leaves were used in the quantification of percentage of lesion types, and three biological replications were conducted.

To further analyse the effect of *MoPCE1* deletion on the pathogenicity of *M. oryzae* , we performed punch inoculation. This method is considered to be more suitable to assess the basal resistance levels of the transgenic plants than the standard spraying inoculation method (Ono et al. 2001; Park et al. 2012). Spore suspension of strains indicated was adjusted to 5 × 10^5^ spores mL^−1^ using sterilised water containing 0.02% vol/vol Tween 20. A 10 μL spore solution was inoculated into punched rice leaves, and the inoculated rice plants were placed in a greenhouse. Disease symptoms were recorded 10 days after inoculation. DNA extraction was performed via the CTAB method using inoculated leaves. The relative fungal biomass [2 ^(*C*_t__*OsACTIN* − *C*_t__*MoPot2*)] were quantified by quantitative real‐time PCR using the primer pairs MoPot2‐qRT‐F/R and OsUG‐qRT‐F/R (Han et al. 2019).

For live‐cell imaging assays, spore solution (5 × 10^5^ spores mL^−1^ in sterilised water) of WT, Δ*Mopce1*, and the complemented strains were inoculated into sheaths of approximately 4‐week‐old rice plants, which were then kept in darkness and high humidity. The hyphal invasion efficiency was investigated 24 h after inoculation using an A1R laser scanning confocal microscope system (Nikon).

### 4.5 Functional Analysis of SP

To verify the function of the MoPce1 SP, we integrated the SP sequence into the pSUC2 vector (which can synthesise tryptophan and carries a sucrose utilisation gene fragment lacking an SP). The constructed vector pSUC2‐MoPCE1, the negative control vector pSUC2‐EV, and the positive control vector pSUC2‐Avr1B were transformed into the yeast strain YTK12 (a nutritional‐deficient strain lacking the sucrose utilisation gene and unable to grow in the absence of tryptophan) using the lithium acetate method. The transformed yeast was plated on CMD−W (tryptophan‐deficient plate) and incubated in darkness at 30°C for 3 days. Then, single colonies grown on CMD−W plate were transferred to a YPRAA plate (10 g L^−1^ yeast extract, 20 g L^−1^ peptone, 20 g L^−1^ sucrose, supplemented with 20 μg mL^−1^ agar) as a carbon source. When an SP with secretion function is connected to the pSUC2 vector, it allows YTK12 strain to grow on the YPRAA plate. The activity of sucrose utilisation enzyme was determined by reducing 2,3,5‐triphenyltetrazolium chloride (TTC) to insoluble red 1,3,5‐triphenylformazan (TPF) (Yin et al. 2018; Xu et al. 2019).

### 4.6 Transient Expression in *N. benthamiana* Leaves

The coding sequence of *MoPCE1* lacking the signal peptide (*MoPCE1* ^*ΔSP*^) was amplified using Pcxsn‐MoPce1‐F/R and inserted into the pCXSN vector, and the constructed vector was introduced into *Agrobacterium tumefaciens* GV3101 competent cells. After the screening on Luria Bertani (LB) agar plates supplemented with 200 μg mL^−1^ kanamycin and 100 μg mL^−1^ rifampicin, the colonies were validated through PCR‐based genotyping using the primers PCXSN‐seq‐F/R (Table S15).

The preparation of inoculum was performed as described previously (Chen, Pan, et al. 2023). In brief, transformed strains were inoculated into liquid LB medium supplemented with 200 μg mL^−1^ kanamycin and 100 μg mL^−1^ rifampicin for 24–36 h. Bacteria were collected and suspended in the *Agrobacterium* infiltration buffer (10 mM MES, 10 mM magnesium chloride, 150 μM acetosyringone; OD_600_ = 0.4). The bacterial suspension was incubated in a culture chamber at 28°C for 4 h, then infiltrated into 4‐week‐old *N. benthamiana* leaves. The inoculated seedlings were cultured in the dark for 48 h. Microscopic examination was performed using an A1R laser scanning confocal microscope system (Nikon).

### 4.7 Transformation of Rice Protoplasts

The transformation of rice protoplasts was performed as described previously (He et al. 2016). Seedlings of the rice cultivar Nipponbare (NPB) were grown in dark at 27°C and 50% relative humidity for about 10 days. The shoots were then cut into 0.5 mm sections and placed in a sterile conical flask containing precooled 0.6 M mannitol. The cut tissues were subjected to vacuum infiltration in the dark for 20 min, and then the supernatant was discarded. Next, 20 mL of enzyme solution (0.6 M mannitol, 10 mM MES, 1% cellulase RS, 0.5% pectolyase R10, 0.1% bovine serum albumin, 1 mM calcium chloride) was added to the conical flask, and the tissues were subjected to vacuum infiltration in the dark for 15 min. The flask was then incubated at 28°C with shaking at 70 rpm for 4 h to allow protoplast release. The enzyme solution was then discarded, and the rice tissues were resuspended in 20 mL of W5 medium (154 mM NaCl, 125 mM CaCl_2_, 5 mM KCl, 5 mM glucose, 2 mM MES, pH adjusted to 5.7 with KOH) to release the protoplasts. The rice tissues were filtered through Miracloth, and the collected rice protoplasts were transferred to a 50 mL EP tube. The plant tissue was washed once with W5 medium, and then the tube was centrifuged at 4°C, 790 *g* to collect the pellet. The pellet was washed once with W5 medium, and the supernatant was discarded. The protoplasts were resuspended in W5 medium to a concentration of 2.5 × 10^6^ cells mL^−1^. The protoplasts were then divided into 2.0 mL centrifuge tubes, with 150 μL per tube, and incubated on ice for 30 min before transformation.

For the transformation, 10 ng of plasmid (pHF223‐GFP‐MoPCE1) was added to 150 μL of protoplasts and gently mixed. An equal volume of 40% PEG was then added, and the mixture was gently inverted. The rice protoplasts were incubated in a 25°C incubator in the dark for 10 min, and then the mixture was diluted with double volume of W5 medium. After centrifugation at 790 *g* for 3 min, the supernatant was discarded, and 500 μL of W5 medium was added. The protoplasts were incubated at 25°C in the dark for 16 h, and the localisation was observed under an A1R laser scanning confocal microscope system (Nikon).

### 4.8 Construction and Validation of Transgenic Rice Plants

The transgenic rice plants ectopically expressing *MoPCE1* ^*ΔSP*^ *‐GFP* fusion in the ZH11 background (*MoPCE1* ^*ΔSP*^ *‐OX*) were constructed by Wuhan Boyuan Company. The ZH11 wild type and *MoPCE1* ^*ΔSP*^ *‐OX* transgenic lines were grown in a greenhouse at 27°C and 50% relative humidity for about 14 days, until the tillering stage. Plant DNA was extracted from its leaves using the CTAB method. A small amount of plant leaves was placed in a 2 mL EP tube with steel beads and frozen in liquid nitrogen. The sample was then ground using a tissue grinder (TISSUELYSER‐2 L, Shanghai Jingxin Industrial Development Co. Ltd.) at 45 Hz for 90 s, with a repeated grinding step. Next, 500 μL of preheated CTAB extraction buffer (2% CTAB, 1.42 M NaCl, 20 mM EDTA, 100 mM Tris‐HCl) and 6.5 μL of RNase were added to each tube, followed by 1 min of shaking and 10–15 min of incubation at 65°C. After that, 0.7 times the volume of chloroform was added, and the mixture was vigorously shaken for 1 min. The sample was then centrifuged at 12,000 *g* for 10 min, and 350 μL of the supernatant was transferred to a new 1.5 mL EP tube. An equal volume of isopropanol was added, and the mixture was incubated at −20°C for 2 h. After centrifugation at 12,000 *g* for 10 min, the supernatant was discarded, and the precipitate was washed with 70% ethanol. Finally, the precipitate was dissolved in 50 μL of double‐distilled water using the MoPCE1‐tF/tR primers for preliminary verification.

For protein extraction, an appropriate amount of leaves was frozen in liquid nitrogen and stored at −80°C. The leaves were ground into powder in liquid nitrogen, and the powder was transferred to a 15 mL EP tube. An appropriate amount of protein lysis buffer (50 mL, lysis buffer: 500 μL PMSF + 500 μL protease inhibitor) was added, mixed thoroughly, and placed on ice for 30 min. The sample was inverted every 10 min to promote lysis. Then, the sample was centrifuged at 4°C, 12,000 *g* for 10 min, and the supernatant was filtered into a new EP tube. 120 μL of the supernatant was taken as the input sample. 20 μL of GFP beads (Tiandi Renhe, Smart‐Lifesciences) was added to a 1.5 mL EP tube, and each tube of beads was washed with 500 μL of washing buffer for three times. The washed beads were transferred to the protein solution and incubated at 4°C with rotation for 3 h. The beads were then captured using a magnetic rack, and the supernatant was removed. The beads were washed with 500 μL of washing buffer for three times. After that, 80 μL of washing buffer and the corresponding 5 × loading buffer were added, and the mixture was boiled at 100°C for 10 min. Finally, western blot analysis was performed using anti‐GFP antibody (M20004; Abmart), anti‐plant actin antibody (ABL1055; Abbkine Scientific), and goat anti‐mouse IgG horseradish peroxidase (HRP) (M21001; Abmart). Western Bright ECL HRP substrate (Advansta) was used for the detection of chemiluminescent signals.

### 4.9 Measurement of ROS in Rice Leaves

The ZH11 wild type and *MoPCE1* ^*ΔSP*^ *‐OX* transgenic lines were grown in a greenhouse at 27°C and 50% relative humidity for about 14 days, until the tillering stage. Rice leaf samples from approximately 6‐week‐old transgenic rice plants were punched with a hole puncher to obtain 2 mm diameter rice discs (taking care to avoid secondary damage to the rice leaves during punching). The prepared rice discs were placed in sterile water and incubated in the dark for 10 h. The rice discs were then placed in a 96‐well plate, with two rice discs per well, and 90 μL of a working solution containing 100 μM luminol, 5 μg mL^−1^ of HRP, and 8 nM hexa‐*N*‐acetyl‐chito‐hexaose. The samples were incubated in the dark for 30 min, and then 10 μL of 500 nM flg22 or 80 nM chitin was added to each well, with water as a control. The production of ROS in the rice tissues was immediately measured using a luminometer (Thermo Scientific) (Park et al. 2012).

### 4.10 Yeast Two‐Hybrid Assay

To validate protein–protein interactions, the constructed plasmids pGBKT7‐MoPCE1 (BD‐MoPce1) and pGADT7‐OsCATC (AD‐OsCATC) were co‐transformed into the yeast strain AH109. The pGBKT7‐p53 (BD‐p53)/pGADT7‐T (AD) and pGBKT7‐Lam (BD‐p53)/pGADT7‐T (AD) were used as positive and negative interaction control pairs, respectively. The candidate clones were grown on SD/−His/−Leu/−Trp/−Ade medium and supplemented with X‐α‐Gal for β‐galactosidase analysis (Miao et al. 2023).

### 4.11 Luciferase Complementation Assay

The coding sequences (CDS) of MoPCE1^Δsp^ and OsCATC were cloned into the pCAMBIA1300‐Cluc and pCAMBIA1300‐NLuc vectors, respectively and transformed into *A. tumefaciens* GV3101 competent cells. The CLuc‐APIP5/AvrPizt‐NLuc pair was used as a positive control (Wang et al. 2016), while the CLuc‐MoPce1^Δsp^/AvrPizt‐NLuc and OsCATC‐NLuc/CLuc‐APIP5 pairs were used as negative controls. The transformed agrobacteria were cultured in 8 mL LB liquid medium containing appropriate antibiotics (50 mg L^−1^ kanamycin + 50 mg L^−1^ rifampicin) at 28°C with shaking (200 rpm) for 36–48 h. Bacterial cells were collected by centrifugation (5000 *g*, 5 min) and resuspended in infiltration buffer (10 mM MES, 10 mM MgCl_2_, 200 μM acetosyringone, pH 5.7) to an OD_600_ of 1.0. Equal volumes of bacterial suspensions were mixed according to experimental combinations. Healthy 4‐week‐old *N*. *benthamiana* plants were selected for transient transformation. The abaxial side of leaves was punctured with sterile needles to create microwounds, and the mixed bacterial suspensions were infiltrated using a 1 mL sterile syringe. Infiltrated areas were marked and subjected to 12 h dark incubation at room temperature, followed by 48 h cultivation under normal light conditions. For luciferase signal detection, 15 μL of 1 mM luciferin solution was evenly applied to the abaxial surface of the infiltrated leaf regions. After 10 min of dark adaptation, luminescence signals were captured using a cooled CCD camera‐based in vivo imaging system (Zhou et al. 2018).

### 4.12 Statistical Analysis

The statistical analysis was performed using the one‐way or two‐way ANOVA, as specified for each experiment, followed by Dunnett's multiple comparisons test, with the indicated control groups, using GraphPad Prism 10 (GraphPad Software; www.graphpad.com).

## Author Contributions

**Zhenhui Zhong:** software, supervision.

## Funding

This research was funded by grants from the National Natural Science Foundation of China (32272513 and 32172365), Natural Science foundation of Fujian Province (2024 J01427), Central Guidance on Local Science and Technology Development Fund of Fujian Province (2022 L3088), and the Postdoctoral Fellowship Program of CPSF (GZC20230448).

## Conflicts of Interest

The authors declare no conflicts of interest.

## Supporting information


**Figure S1:** Deletion of *MoPCE1* compromised the pathogenicity of rice blast fungus. (A) Strategy for the generation of *MoPCE1*. (B) Evaluation of Δ*Mopce1* candidates through Southern blot. The arrow heads indicate the target band predicted for Guy11 wild type (3.1 kb) and the Δ*Mopce1* strains (5.2 kb).

**Figure S2:** MoPce1 is a conserved CAP protein. (A) Multiple sequence alignment of MoPce1 and CAP proteins from *F. graminearum* (FGSG_03109), *C. chrysosperma* (CcCap2), *S. cerevisiae* (PRY1), *A. thaliana* (PR‐1), and *U. maydis* (UMAG_01204). The four α‐helices (α1‐4) and three β‐sheet (β1‐3) were marked with blue lines; red arrows indicate the conserved cysteine residuals. (B) Phylogenetic tree was constructed using the CAP homologues from *C. chrysosperma* (CcCAP1‐3), *Valsa malicola* (VMCG_03776), *F. oxysporum* (FOZG_13166, FOXG_09795, FOXG_06245, FOXG_10300, FOXG_12292 and FOXG_14109), *C. albicans* (Rbt4, CAALFM_C110810WA, CAALFM_C107580CA, CAALFM_C114120CA and CAALFM_C107040CA), *S. cerevisiae* (PRY1‐3), *Solanum lycopersicum* (P14a), *A. thaliana* (PR‐1), *N. tabacum* (PR1a, PR1b and PR1c), *Oryza sativa* (Os01g0971100), *Neurospora crassa* (NCU_02470, NCU_05618), *Aspergillus fumigatus* (Afu1g02040, and Afu1g12350), *Aspergillus nidulans* (AN1058 and AN10057), *Botrytis cinerea* (BCIG_08280 and BCIG_09594), *F. graminearum* (FGSG_02744, FGSG_09548, FGSG_03109 and FGSG_03312), *M. oryzae* (MGG_07807, MGG_03085, MGG_13936 and MGG_03755), *U. maydis* (UMAG_01204 and UMAG_04343), and *Coccidioides immitis* (CIMG_09974 and CIMG_06897).

**Figure S3:** MoPce1 is dispensable for asexual development of *M. oryzae* . (A,B) Colony morphology (A) and diameter (B) of Guy11 wild type, Δ*Mopce1*, and complemented strains grown on CM plates. No significant difference was detected. (C) Number of conidia collected from a 7 cm rice bran plate at 3 days after induction of conidiation. No significant difference was detected.

**Figure S4:** The GFP fusions of MoPce1 were functional. (A‐C) The morphology (A), size (B) and relative biomass (C) of lesions caused by Guy11 wild type, Δ*Mopce1*, and Δ*Mopce1* strains ectopically expressing the *MoPCE1‐GFP* (Δ*Mopce1*/ *MoPCE1‐GFP*) or *GFP‐MoPCE1* (Δ*Mopce1*/ *GFP‐MoPCE1*). The leaves were photographed 10 days after inoculation. Statistical analysis was performed using one‐way ANOVA followed by Dunnett's multiple comparisons test, with Guy11 as the control. The data are shown as means ± standard error (*n* = 6). ****p < 0.0001.

**Figure S5:** Mapping of the fragment required for the interaction between OsCATC and MoPce1. (A) Schematic diagram of truncated OsCATC protein constructs. For mapping of fragment involved in the interaction between OsCATC and MoPce1, the OsCATC was divided into three fragments: N fragment (1–230 aa), C1 fragment (231–360 aa) and C2 fragment (361–492 aa). (B) Yeast two‐hybrid (Y2H) assay validating the involvement of OsCATC_C1 in the interaction between OsCATC and MoPce1.

**Table S1:** List of putative core effectors.

**Table S2:** List of PCEs functionally analysed.

**Table S3:** The colony diameter of Δ*Mopce1* strain.

**Table S4:** The conidiation of Δ*Mopce1* strain.

**Table S5:** The percentage of lesion types of Δ*Mopce1* strain.

**Table S6:** The lesion size caused by Δ*Mopce1* strains ectopically expressed the green fluorescence protein (GFP) fused *MoPCE1*.

**Table S7:** The relative biomass of lesions caused by Δ*Mopce1* strains ectopically expressed the green fluorescence protein (GFP) fused *MoPCE1*.

**Table S8:** The relative expression level of MoPCE^Δsp^ in transgenic plants.

**Table S9:** The lesion size on MoPCE^Δsp^‐OX transgenic plants caused by *M. oryzae* inoculation.

**Table S10:** The relative biomass of lesions on MoPCE^Δsp^‐OX transgenic plants caused by *M. oryzae* inoculation.

**Table S11:** The luminescence generated from the wild type and MoPCE^Δsp^‐OX transgenic plants in response to flg22.

**Table S12:** The luminescence generated from the wild type and MoPCE^Δsp^‐OX transgenic plants in response to Chitin.

**Table S13:** The relative expression level of PR genes in the MoPCE^Δsp^‐OX transgenic plants challenged with or without *M. oryzae* .

**Table S14:** The list of putative MoPce1 interacting proteins screened through Yeast‐two‐hybrid.

**Table S15:** The lesion size on *oscatc* transgenic plants caused by *M. oryzae* inoculation.

**Table S16:** The relative biomass of lesions on *oscatc* transgenic plants caused by *M. oryzae* inoculation.

**Table S17:** The luminescence generated from the wild type and *oscatc* plants in response to flg22.

**Table S18:** The luminescence generated from the wild type and *oscatc* plants in response to Chitin.

**Table S19:** Primers used in this study.

## Data Availability

The data that supports the findings of this study are available in the Supporting information of this article.

## References

[mpp70206-bib-0001] Abraham, A. , and D. E. Chandler . 2017. “Tracing the Evolutionary History of the CAP Superfamily of Proteins Using Amino Acid Sequence Homology and Conservation of Splice Sites.” Journal of Molecular Evolution 85: 137–157. 29071358 10.1007/s00239-017-9813-9

[mpp70206-bib-0002] Bakkali, F. , S. Averbeck , D. Averbeck , and M. Idaomar . 2008. “Biological Effects of Essential Oils – A Review.” Food and Chemical Toxicology 46: 446–475. 17996351 10.1016/j.fct.2007.09.106

[mpp70206-bib-0003] Braun, B. R. , W. S. Head , M. X. Wang , and A. D. Johnson . 2000. “Identification and Characterization of TUP1‐Regulated Genes in *Candida albicans* .” Genetics 156: 31–44. 10978273 10.1093/genetics/156.1.31 PMC1461230

[mpp70206-bib-0004] Chen, H. , R. King , D. Smith , et al. 2023. “Combined Pangenomics and Transcriptomics Reveals Core and Redundant Virulence Processes in a Rapidly Evolving Fungal Plant Pathogen.” BMC Biology 21: 24. 36747219 10.1186/s12915-023-01520-6 PMC9903594

[mpp70206-bib-0005] Chen, S. , P. Songkumarn , R. C. Venu , et al. 2013. “Identification and Characterization of in Planta‐Expressed Secreted Effector Proteins From *Magnaporthe oryzae* That Induce Cell Death in Rice.” Molecular Plant–Microbe Interactions 26: 191–202. 23035914 10.1094/MPMI-05-12-0117-R

[mpp70206-bib-0006] Chen, X. , S. Pan , H. Bai , et al. 2023. “A Nonclassically Secreted Effector of *Magnaporthe oryzae* Targets Host Nuclei and Plays Important Roles in Fungal Growth and Plant Infection.” Molecular Plant Pathology 24: 1093–1106. 37306516 10.1111/mpp.13356 PMC10423324

[mpp70206-bib-0007] Choudhary, V. , and R. Schneiter . 2012. “Pathogen‐Related Yeast (PRY) Proteins and Members of the CAP Superfamily Are Secreted Sterol‐Binding Proteins.” Proceedings of the National Academy of Sciences of the United States of America 109: 16882–16887. 23027975 10.1073/pnas.1209086109 PMC3479496

[mpp70206-bib-0008] Consortium . 2015. “UniProt: A Hub for Protein Information.” Nucleic Acids Research 43: D204–D212. 25348405 10.1093/nar/gku989 PMC4384041

[mpp70206-bib-0009] Cottier, S. , R. Darwiche , F. Meyenhofer , M. O. Debelyy , and R. Schneiter . 2020. “The Yeast Cell Wall Protein Pry3 Inhibits Mating Through Highly Conserved Residues Within the CAP Domain.” Biology Open 9: bio053470. 32554483 10.1242/bio.053470 PMC7340583

[mpp70206-bib-0010] Darwiche, R. , O. El Atab , R. M. Baroni , et al. 2017. “Plant Pathogenesis‐Related Proteins of the Cacao Fungal Pathogen *Moniliophthora perniciosa* Differ in Their Lipid‐Binding Specificities.” Journal of Biological Chemistry 292: 20558–20569. 29042440 10.1074/jbc.M117.811398 PMC5733592

[mpp70206-bib-0011] Emms, D. M. , and S. Kelly . 2015. “OrthoFinder: Solving Fundamental Biases in Whole Genome Comparisons Dramatically Improves Orthogroup Inference Accuracy.” Genome Biology 16: 157. 26243257 10.1186/s13059-015-0721-2 PMC4531804

[mpp70206-bib-0012] Gamir, J. , R. Darwiche , P. Van't Hof , et al. 2017. “The Sterol‐Binding Activity of PATHOGENESIS‐RELATED PROTEIN 1 Reveals the Mode of Action of an Antimicrobial Protein.” Plant Journal 89: 502–509. 10.1111/tpj.13398 27747953

[mpp70206-bib-0013] Gibbs, G. M. , K. Roelants , and M. K. O'Bryan . 2008. “The CAP Superfamily: Cysteine‐Rich Secretory Proteins, Antigen 5, and Pathogenesis‐Related 1 Proteins‐Roles in Reproduction, Cancer, and Immune Defense.” Endocrine Reviews 29: 865–897. 18824526 10.1210/er.2008-0032

[mpp70206-bib-0014] Giraldo, M. C. , and B. Valent . 2013. “Filamentous Plant Pathogen Effectors in Action.” Nature Reviews Microbiology 11: 800–814. 24129511 10.1038/nrmicro3119

[mpp70206-bib-0015] Han, Y. , L. Song , C. Peng , et al. 2019. “A *Magnaporthe* Chitinase Interacts With a Rice Jacalin‐Related Lectin to Promote Host Colonization.” Plant Physiology 179: 1416–1430. 30696749 10.1104/pp.18.01594 PMC6446787

[mpp70206-bib-0016] Han, Z. , D. Xiong , R. Schneiter , and C. Tian . 2023. “The Function of Plant PR1 and Other Members of the CAP Protein Superfamily in Plant–Pathogen Interactions.” Molecular Plant Pathology 24: 651–668. 36932700 10.1111/mpp.13320 PMC10189770

[mpp70206-bib-0017] Han, Z. , D. Xiong , Z. Xu , T. Liu , and C. Tian . 2021. “The *Cytospora chrysosperma* Virulence Effector CcCAP1 Mainly Localizes to the Plant Nucleus to Suppress Plant Immune Responses.” mSphere 6: e00883‐20. 33627507 10.1128/mSphere.00883-20 PMC8544888

[mpp70206-bib-0018] He, F. , S. Chen , Y. Ning , and G. L. Wang . 2016. “Rice ( *Oryza sativa* ) Protoplast Isolation and Its Application for Transient Expression Analysis.” Current Protocols in Plant Biology 1: 373–383. 30775867 10.1002/cppb.20026

[mpp70206-bib-0019] Hoang, C. V. , C. K. Bhaskar , and L. S. Ma . 2021. “A Novel Core Effector Vp1 Promotes Fungal Colonization and Virulence of *Ustilago maydis* .” Journal of Fungi (Basel) 7: 589. 10.3390/jof7080589 PMC8396986 34436129

[mpp70206-bib-0020] Hu, L. , Y. Yang , L. Jiang , and S. Liu . 2016. “The Catalase Gene Family in Cucumber: Genome‐Wide Identification and Organization.” Genetics and Molecular Biology 39: 408–415. 27560990 10.1590/1678-4685-GMB-2015-0192 PMC5004828

[mpp70206-bib-0021] Irieda, H. , Y. Inoue , M. Mori , et al. 2019. “Conserved Fungal Effector Suppresses PAMP‐Triggered Immunity by Targeting Plant Immune Kinases.” Proceedings of the National Academy of Sciences of the United States of America 116: 496–505. 30584105 10.1073/pnas.1807297116 PMC6329965

[mpp70206-bib-0022] Jones, J. D. G. , and J. L. Dangl . 2006. “The Plant Immune System.” Nature 444: 323–329. 17108957 10.1038/nature05286

[mpp70206-bib-0023] Khang, C.‐H. , E. Richardson , Y. Hernandez‐Rodriguez , L. Chen , T. Todd , and B. Valent . 2013. “Trafficking Mechanism of Fungal Effector Proteins Inside Rice Cells.” Microscopy and Microanalysis 19: 210–211.23920208

[mpp70206-bib-0024] Liao, Y. , A. Ali , Z. Xue , et al. 2022. “Disruption of LLM9428/OsCATC Represses Starch Metabolism and Confers Enhanced Blast Resistance in Rice.” 23: 3827. 10.3390/ijms23073827 PMC8998287 35409186

[mpp70206-bib-0025] Lin, A. , Y. Wang , J. Tang , et al. 2011. “Nitric Oxide and Protein S‐Nitrosylation Are Integral to Hydrogen Peroxide‐Induced Leaf Cell Death in Rice.” Plant Physiology 158: 451–464. 22106097 10.1104/pp.111.184531 PMC3252116

[mpp70206-bib-0026] Lin, Y. H. , M. Y. Xu , C. C. Hsu , et al. 2023. “ *Ustilago maydis* PR‐1‐Like Protein Has Evolved Two Distinct Domains for Dual Virulence Activities.” Nature Communications 14: 5755. 10.1038/s41467-023-41459-4 PMC10505147 37716995

[mpp70206-bib-0027] Liu, Y. , T. Sun , Y. Li , et al. 2025. “Proteomic Analysis Revealed the Function of PoElp3 in Development, Pathogenicity, and Autophagy Through the tRNA‐Mediated Translation Efficiency in the Rice Blast Fungus.” Journal of Integrative Agriculture 24: 1515–1528.

[mpp70206-bib-0028] Lozano‐Torres, J. L. , R. H. Wilbers , P. Gawronski , et al. 2012. “Dual Disease Resistance Mediated by the Immune Receptor Cf‐2 in Tomato Requires a Common Virulence Target of a Fungus and a Nematode.” Proceedings of the National Academy of Sciences of the United States of America 109: 10119–10124. 22675118 10.1073/pnas.1202867109 PMC3382537

[mpp70206-bib-0029] Lozano‐Torres, J. L. , R. H. P. Wilbers , S. Warmerdam , et al. 2014. “Apoplastic Venom Allergen‐Like Proteins of Cyst Nematodes Modulate the Activation of Basal Plant Innate Immunity by Cell Surface Receptors.” PLoS Pathogens 10: e1004569. 25500833 10.1371/journal.ppat.1004569 PMC4263768

[mpp70206-bib-0030] Lu, S. , and M. C. Edwards . 2018. “Molecular Characterization and Functional Analysis of PR‐1‐Like Proteins Identified From the Wheat Head Blight Fungus *Fusarium graminearum* .” Phytopathology 108: 510–520. 29117786 10.1094/PHYTO-08-17-0268-R

[mpp70206-bib-0031] Luo, X. , T. Tian , L. Feng , et al. 2023. “Pathogenesis‐Related Protein 1 Suppresses Oomycete Pathogen by Targeting Against AMPK Kinase Complex.” Journal of Advanced Research 43: 13–26. 36585103 10.1016/j.jare.2022.02.002 PMC9811325

[mpp70206-bib-0032] Mentlak, T. A. , A. Kombrink , T. Shinya , et al. 2012. “Effector‐Mediated Suppression of Chitin‐Triggered Immunity by *Magnaporthe oryzae* Is Necessary for Rice Blast Disease.” Plant Cell 24: 322–335. 22267486 10.1105/tpc.111.092957 PMC3289562

[mpp70206-bib-0033] Miao, P. , X. Mao , S. Chen , et al. 2023. “The Mitotic Exit Mediated by Small GTPase Tem1 Is Essential for the Pathogenicity of *Fusarium graminearum* .” PLoS Pathogens 19: e1011255. 36928713 10.1371/journal.ppat.1011255 PMC10047555

[mpp70206-bib-0034] Morgan, W. , and S. Kamoun . 2007. “RXLR Effectors of Plant Pathogenic Oomycetes.” Current Opinion in Microbiology 10: 332–338. 17707688 10.1016/j.mib.2007.04.005

[mpp70206-bib-0035] Mosquera, G. , M. C. Giraldo , C. H. Khang , S. Coughlan , and B. Valent . 2009. “Interaction Transcriptome Analysis Identifies *Magnaporthe oryzae* BAS1‐4 as Biotrophy‐Associated Secreted Proteins in Rice Blast Disease.” Plant Cell 21: 1273–1290. 19357089 10.1105/tpc.107.055228 PMC2685627

[mpp70206-bib-0036] Ono, E. , H. L. Wong , T. Kawasaki , M. Hasegawa , O. Kodama , and K. Shimamoto . 2001. “Essential Role of the Small GTPase Rac in Disease Resistance of Rice.” Proceedings of the National Academy of Sciences of the United States of America 98: 759–764. 11149940 10.1073/pnas.021273498 PMC14661

[mpp70206-bib-0037] Park, C. H. , S. Chen , G. Shirsekar , et al. 2012. “The *Magnaporthe oryzae* Effector *AvrPiz‐t* Targets the RING E3 Ubiquitin Ligase APIP6 to Suppress Pathogen‐Associated Molecular Pattern‐Triggered Immunity in Rice.” Plant Cell 24: 4748–4762. 23204406 10.1105/tpc.112.105429 PMC3531864

[mpp70206-bib-0038] Prados‐Rosales, R. C. , R. Roldán‐Rodríguez , C. Serena , et al. 2012. “A PR‐1‐Like Protein of *Fusarium oxysporum* Functions in Virulence on Mammalian Hosts.” Journal of Biological Chemistry 287: 21970–21979. 22553200 10.1074/jbc.M112.364034 PMC3381157

[mpp70206-bib-0039] Röhm, M. , E. Lindemann , E. Hiller , et al. 2013. “A Family of Secreted Pathogenesis‐Related Proteins in *Candida albicans* .” Molecular Microbiology 87: 132–151. 23136884 10.1111/mmi.12087

[mpp70206-bib-0040] Saitoh, H. , S. Fujisawa , C. Mitsuoka , et al. 2012. “Large‐Scale Gene Disruption in *Magnaporthe oryzae* Identifies MC69, a Secreted Protein Required for Infection by Monocot and Dicot Fungal Pathogens.” PLoS Pathogens 8: e1002711. 22589729 10.1371/journal.ppat.1002711 PMC3349759

[mpp70206-bib-0041] Schneiter, R. , and A. Di Pietro . 2013. “The CAP Protein Superfamily: Function in Sterol Export and Fungal Virulence.” Biomolecular Concepts 4: 519–525. 25436594 10.1515/bmc-2013-0021

[mpp70206-bib-0042] Teixeira, P. J. , D. P. Thomazella , R. O. Vidal , et al. 2012. “The Fungal Pathogen *Moniliophthora perniciosa* Has Genes Similar to Plant PR‐1 That Are Highly Expressed During Its Interaction With Cacao.” PLoS One 7: e45929. 23029323 10.1371/journal.pone.0045929 PMC3447762

[mpp70206-bib-0043] Valent, B. 2021. “The Impact of Blast Disease: Past, Present, and Future.” Methods in Molecular Biology 2356: 1–18. 34236673 10.1007/978-1-0716-1613-0_1

[mpp70206-bib-0044] Wang, B. , P. Xue , Y. Zhang , et al. 2024. “OsCPK12 Phosphorylates OsCATA and OsCATC to Regulate H_2_O_2_ Homeostasis and Improve Oxidative Stress Tolerance in Rice.” Plant Communications 5: 100780. 38130060 10.1016/j.xplc.2023.100780 PMC10943579

[mpp70206-bib-0045] Wang, R. , Y. Ning , X. Shi , et al. 2016. “Immunity to Rice Blast Disease by Suppression of Effector‐Triggered Necrosis.” Current Biology 16: 2399–2411. 10.1016/j.cub.2016.06.072 27641772

[mpp70206-bib-0046] Xu, Q. , C. Tang , X. Wang , et al. 2019. “An Effector Protein of the Wheat Stripe Rust Fungus Targets Chloroplasts and Suppresses Chloroplast Function.” Nature Communications 10: 5571. 10.1038/s41467-019-13487-6 PMC6895047 31804478

[mpp70206-bib-0047] Yang, T. , and B. W. Poovaiah . 2002. “Hydrogen Peroxide Homeostasis: Activation of Plant Catalase by Calcium/Calmodulin.” Proceedings of the National Academy of Sciences of the United States of America 99: 4097–4102. 11891305 10.1073/pnas.052564899 PMC122654

[mpp70206-bib-0048] Yang, Z. , M. Li , L. Huang , et al. 2024. “MoSey1 Regulates the Unfolded Protein Response, Appressorium Development, and Pathogenicity of *Magnaporthe oryzae* .” Phytopathology Research 6: 33. 10.1186/s42483-024-00253-w.

[mpp70206-bib-0049] Ye, N. , G. Zhu , Y. Liu , Y. Li , and J. Zhang . 2011. “ABA Controls H_2_O_2_ Accumulation Through the Induction of OsCATB in Rice Leaves Under Water Stress.” Plant and Cell Physiology 52: 689–698. 21398647 10.1093/pcp/pcr028

[mpp70206-bib-0050] Yin, W. , Y. Wang , T. Chen , Y. Lin , and C. Luo . 2018. “Functional Evaluation of the Signal Peptides of Secreted Proteins.” Bio‐Protocol 8: e2839. 34286044 10.21769/BioProtoc.2839 PMC8275294

[mpp70206-bib-0051] You, X. , F. Zhang , Z. Liu , et al. 2022. “Rice Catalase OsCATC Is Degraded by E3 Ligase APIP6 to Negatively Regulate Immunity.” Plant Physiology 190: 1095–1099. 35781740 10.1093/plphys/kiac317 PMC9516720

[mpp70206-bib-0052] Yuan, M. , B. P. M. Ngou , P. Ding , and X. F. Xin . 2021. “PTI‐ETI Crosstalk: An Integrative View of Plant Immunity.” Current Opinion in Plant Biology 62: 102030. 33684883 10.1016/j.pbi.2021.102030

[mpp70206-bib-0053] Zhang, H. , M. Mukherjee , J. E. Kim , W. Yu , and W. B. Shim . 2018. “Fsr1, a Striatin Homologue, Forms an Endomembrane‐Associated Complex That Regulates Virulence in the Maize Pathogen *Fusarium verticillioides* .” Molecular Plant Pathology 19: 812–826. 28467007 10.1111/mpp.12562 PMC6638083

[mpp70206-bib-0054] Zhang, H. , J. Yang , M. Liu , et al. 2024. “Early Molecular Events in the Interaction Between *Magnaporthe oryzae* and Rice.” Phytopathology Research 6: 9.

[mpp70206-bib-0055] Zhang, L. M. , S. T. Chen , M. Qi , et al. 2021. “The Putative Elongator Complex Protein Elp3 Is Involved in Asexual Development and Pathogenicity by Regulating Autophagy in the Rice Blast Fungus.” Journal of Integrative Agriculture 20: 2944–2956.

[mpp70206-bib-0056] Zhang, Z. , Y. Xu , Z. Xie , X. Li , Z.‐H. He , and X.‐X. Peng . 2016. “Association–Dissociation of Glycolate Oxidase With Catalase in Rice: A Potential Switch to Modulate Intracellular H_2_O_2_ Levels.” Molecular Plant 9: 737–748. 26900141 10.1016/j.molp.2016.02.002

[mpp70206-bib-0057] Zhao, M. , Y. Zhang , H. Guo , et al. 2023. “Identification and Functional Analysis of CAP Genes From the Wheat Stripe Rust Fungus *Puccinia striiformis* f. sp. *tritici* .” Journal of Fungi (Basel) 9: 734. 10.3390/jof9070734 PMC10381272 37504723

[mpp70206-bib-0058] Zheng, H. , Z. Zhong , M. Shi , et al. 2018. “Comparative Genomic Analysis Revealed Rapid Differentiation in the Pathogenicity‐Related Gene Repertoires Between *Pyricularia oryzae* and *Pyricularia penniseti* Isolated From a *Pennisetum* Grass.” BMC Genomics 19: 927. 30545292 10.1186/s12864-018-5222-8 PMC6293661

[mpp70206-bib-0059] Zhong, Z. , J. Norvienyeku , M. Chen , et al. 2016. “Directional Selection From Host Plants Is a Major Force Driving Host Specificity in *Magnaporthe* Species.” Scientific Reports 6: 25591. 27151494 10.1038/srep25591 PMC4858695

[mpp70206-bib-0060] Zhou, Z. , G. Bi , and J. M. Zhou . 2018. “Luciferase Complementation Assay for Protein–Protein Interactions in Plants.” Current Protocols in Plant Biology 3: 42–50. 30040251 10.1002/cppb.20066

