# Supplementary material for: A Core Effector MoPce1 Is Required for the Pathogenicity of Magnaporthe oryzae by Modulating Catalase‐Mediated H2O2 Homeostasis in Rice
Source: Mol Plant Pathol. 2026 Jan 16;27(1):e70206. doi: 10.1111/mpp.70206 (PMC12811410; doi:10.1111/mpp.70206)
Supplement: Supplementary file 1 — Figure S1: Deletion of MoPCE1 compromised the pathogenicity of rice blast fungus. (A) Strategy for the generation of MoPCE1. (B) Evaluation of ΔMopce1 candidates through Southern blot. The arrow heads indicate the target band predicted for Guy11 wild type (3.1 kb) and the ΔMopce1 strains (5.2 kb). [file MPP-27-e70206-s001.docx]

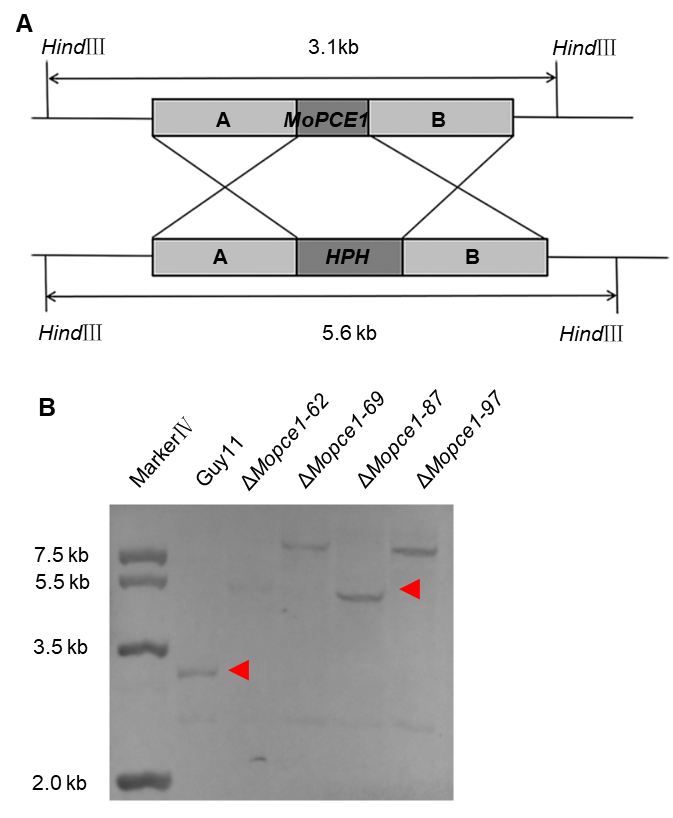


**Figure S1.** Deletion of *MoPCE1* compromised the pathogenicity of rice blast fungus. (A) Strategy for the generation of *MoPCE1*. (B) Evaluation of Δ*Mopce1* candidates through Southern blot. The arrow heads indicate the target band predicted for Guy11 wild type (3.1 kb) and the Δ*Mopce1* strains (5.2 kb).
