# Supplementary material for: A Core Effector MoPce1 Is Required for the Pathogenicity of Magnaporthe oryzae by Modulating Catalase‐Mediated H2O2 Homeostasis in Rice
Source: Mol Plant Pathol. 2026 Jan 16;27(1):e70206. doi: 10.1111/mpp.70206 (PMC12811410; doi:10.1111/mpp.70206)
Supplement: Supplementary file 2 — Figure S2: MoPce1 is a conserved CAP protein. (A) Multiple sequence alignment of MoPce1 and CAP proteins from F. graminearum (FGSG_03109), C. chrysosperma (CcCap2), S. cerevisiae (PRY1), A. thaliana (PR‐1), and U. maydis (UMAG_01204). The four α‐helices (α1‐4) and three β‐sheet (β1‐3) were marked with blue lines; red arrows indicate the conserved cysteine residuals. (B) Phylogenetic tree was constructed using the CAP homologues from C. chrysosperma (CcCAP1‐3), Valsa malicola (VMCG_03776), F. oxysporum (FOZG_13166, FOXG_09795, FOXG_06245, FOXG_10300, FOXG_12292 and FOXG_14109), C. albicans (Rbt4, CAALFM_C110810WA, CAALFM_C107580CA, CAALFM_C114120CA and CAALFM_C107040CA), S. cerevisiae (PRY1‐3), Solanum lycopersicum (P14a), A. thaliana (PR‐1), N. tabacum (PR1a, PR1b and PR1c), Oryza sativa (Os01g0971100), Neurospora crassa (NCU_02470, NCU_05618), Aspergillus fumigatus (Afu1g02040, and Afu1g12350), Aspergillus nidulans (AN1058 and AN10057), Botrytis cinerea (BCIG_08280 and BCIG_09594), F. graminearum (FGSG_02744, FGSG_09548, FGSG_03109 and FGSG_03312), M. oryzae (MGG_07807, MGG_03085, MGG_13936 and MGG_03755), U. maydis (UMAG_01204 and UMAG_04343), and Coccidioides immitis (CIMG_09974 and CIMG_06897). [file MPP-27-e70206-s013.docx]

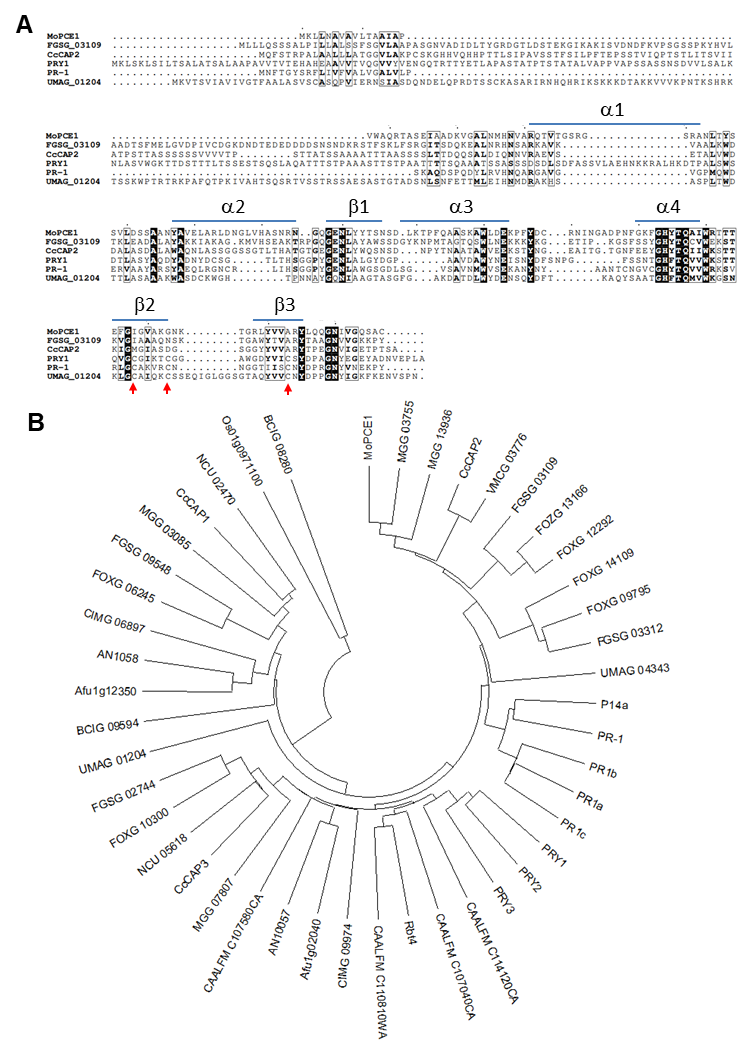


**Figure S2.** MoPce1 is a conserved CAP protein. a Multiple sequence alignment of MoPce1 and CAP proteins from *F. graminearum* (FGSG_03109), *C. chrysosperma* (CcCap2), *S. cerevisiae* (PRY1), *A. thaliana* (PR-1), and *U. maydis* (UMAG_01204). The four α- helices (α1-4) and three β-sheet (β1-3) were marked with blue lines; red arrows indicate the conserved cysteine residuals. b Phylogenetic tree was constructed using the CAP homologs from *C. chrysosperma* (CcCAP1-3), *Valsa malicola* (VMCG_03776), *F. oxysporum* (FOZG_13166, FOXG_09795, FOXG_06245, FOXG_10300, FOXG_12292 and FOXG_14109), *C. albicans* (Rbt4, CAALFM_C110810WA, CAALFM_C107580CA, CAALFM_C114120CA and CAALFM_C107040CA), *S. cerevisiae* (PRY1-3), *Solanum lycopersicum* (P14a), *A. thaliana* (PR-1), *N. tabacum* (PR1a, PR1b and PR1c), *Oryza sativa* (Os01g0971100), *Neurospora crassa* (NCU_02470, NCU_05618), *Aspergillus fumigatus* (Afu1g02040, and Afu1g12350), *Aspergillus nidulans* (AN1058 and AN10057), *Botrytis cinerea* (BCIG_08280 and BCIG_09594), *F. graminearum* (FGSG_02744, FGSG_09548, FGSG_03109 and FGSG_03312), *M. oryzae* (MGG_07807, MGG_03085, MGG_13936 and MGG_03755), *U. maydis* (UMAG_01204 and

UMAG_04343), and *Coccidioides immitis* (CIMG_09974 and CIMG_06897).
