# Supplementary material for: A Core Effector MoPce1 Is Required for the Pathogenicity of Magnaporthe oryzae by Modulating Catalase‐Mediated H2O2 Homeostasis in Rice
Source: Mol Plant Pathol. 2026 Jan 16;27(1):e70206. doi: 10.1111/mpp.70206 (PMC12811410; doi:10.1111/mpp.70206)
Supplement: Supplementary file 3 — Figure S3: MoPce1 is dispensable for asexual development of M. oryzae . (A,B) Colony morphology (A) and diameter (B) of Guy11 wild type, ΔMopce1, and complemented strains grown on CM plates. No significant difference was detected. (C) Number of conidia collected from a 7 cm rice bran plate at 3 days after induction of conidiation. No significant difference was detected. [file MPP-27-e70206-s021.docx]

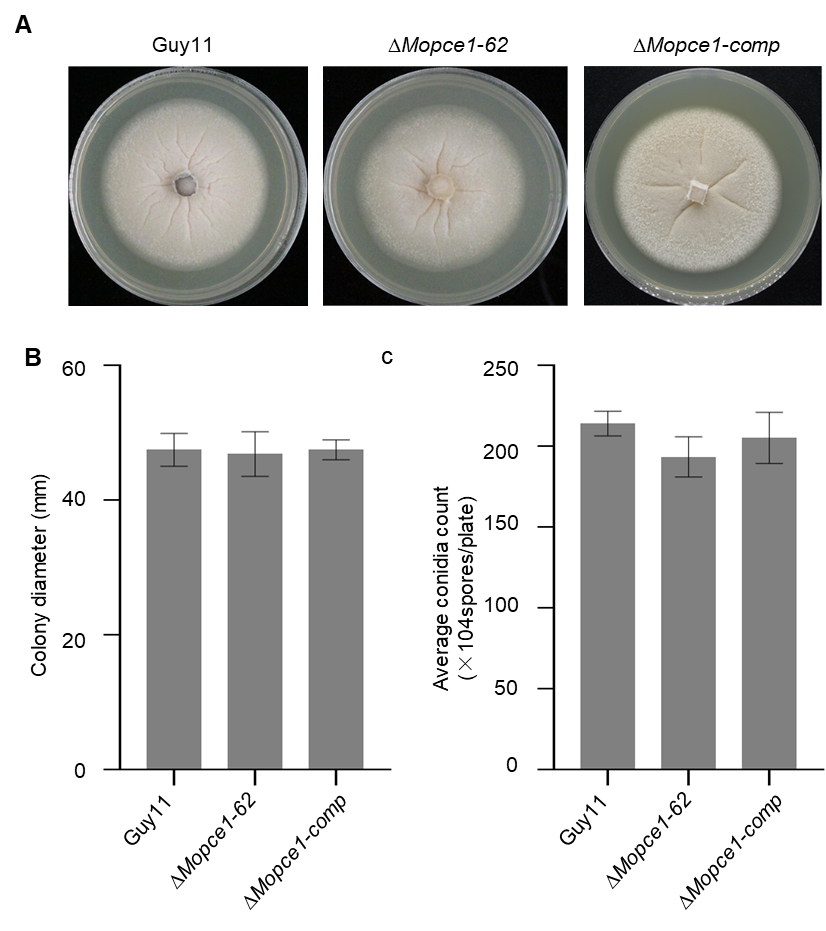


**Figure S3**: MoPce1 is dispensable for asexual development of *M. oryzae*. (A,B) Colony morphology (A) and diameter (B) of Guy11 wild type, Δ*Mopce1*, and complemented strains grown on CM plates. No significant difference was detected. (C) Number of conidia collected from a 7 cm rice bran plate at 3 days after induction of conidiation. No significant difference was detected.
