# Supplementary material for: A Core Effector MoPce1 Is Required for the Pathogenicity of Magnaporthe oryzae by Modulating Catalase‐Mediated H2O2 Homeostasis in Rice
Source: Mol Plant Pathol. 2026 Jan 16;27(1):e70206. doi: 10.1111/mpp.70206 (PMC12811410; doi:10.1111/mpp.70206)
Supplement: Supplementary file 4 — Figure S4: The GFP fusions of MoPce1 were functional. (A‐C) The morphology (A), size (B) and relative biomass (C) of lesions caused by Guy11 wild type, ΔMopce1, and ΔMopce1 strains ectopically expressing the MoPCE1‐GFP (ΔMopce1/ MoPCE1‐GFP) or GFP‐MoPCE1 (ΔMopce1/ GFP‐MoPCE1). The leaves were photographed 10 days after inoculation. Statistical analysis was performed using one‐way ANOVA followed by Dunnett's multiple comparisons test, with Guy11 as the control. The data are shown as means ± standard error (n = 6). ****p < 0.0001. [file MPP-27-e70206-s011.docx]

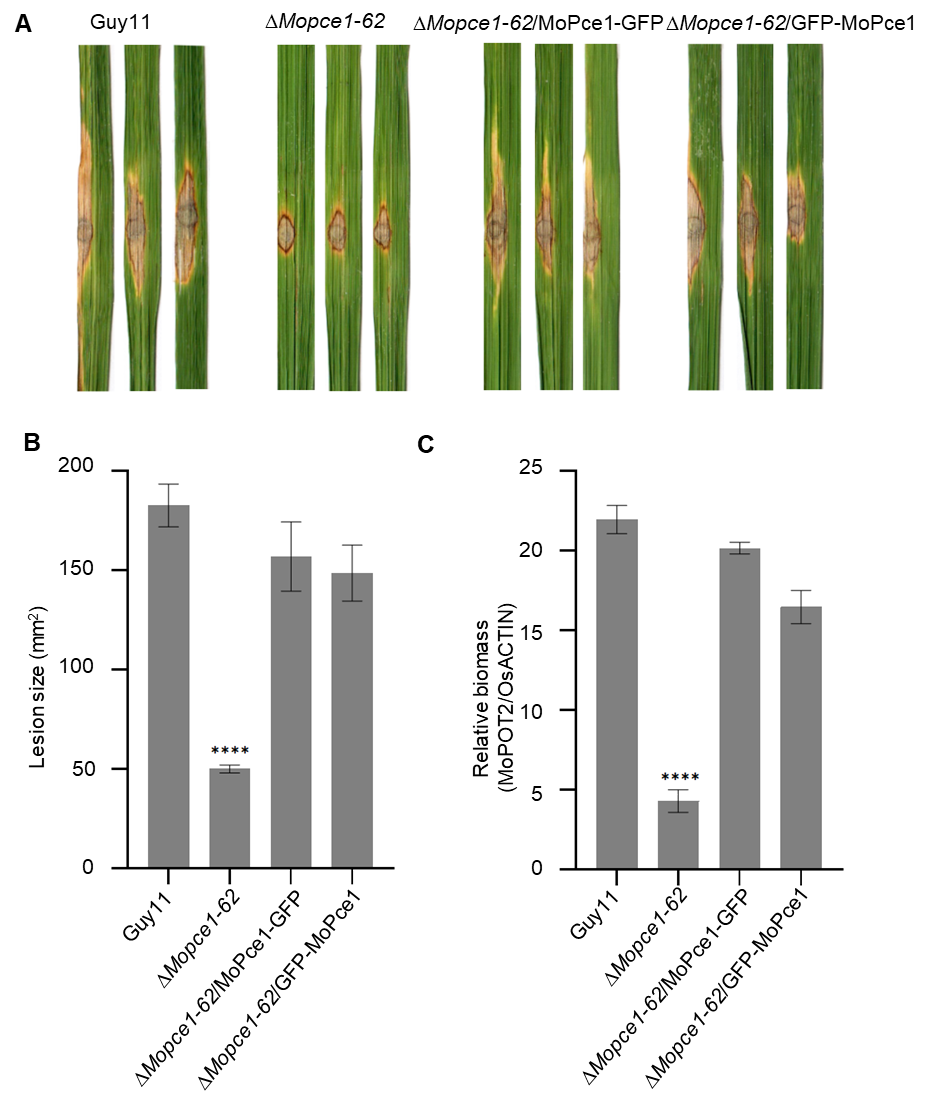


**Figure S4.** The GFP-MoPce1 fusion was functional. (A-C) The morphology (A), size (B) and relative biomass (C) of lesions caused by Guy11 wild type, Δ*Mopce1*, and Δ*Mopce1* strains ectopically expressing the *MoPCE1-GFP* (Δ*Mopce1/* *MoPCE1-GFP*) or *GFP-MoPCE1* (Δ*Mopce1/* *GFP-MoPCE1*). The leaves were photographed 10 days after inoculation. Statistical analysis was performed using one-way ANOVA followed by Dunnett’s multiple comparisons test, with Guy11 as the control. The data are shown as means ± standard error (n = 6). ****p < 0.0001.
