# Supplementary material for: A Core Effector MoPce1 Is Required for the Pathogenicity of Magnaporthe oryzae by Modulating Catalase‐Mediated H2O2 Homeostasis in Rice
Source: Mol Plant Pathol. 2026 Jan 16;27(1):e70206. doi: 10.1111/mpp.70206 (PMC12811410; doi:10.1111/mpp.70206)
Supplement: Supplementary file 5 — Figure S5: Mapping of the fragment required for the interaction between OsCATC and MoPce1. (A) Schematic diagram of truncated OsCATC protein constructs. For mapping of fragment involved in the interaction between OsCATC and MoPce1, the OsCATC was divided into three fragments: N fragment (1–230 aa), C1 fragment (231–360 aa) and C2 fragment (361–492 aa). (B) Yeast two‐hybrid (Y2H) assay validating the involvement of OsCATC_C1 in the interaction between OsCATC and MoPce1. [file MPP-27-e70206-s002.docx]

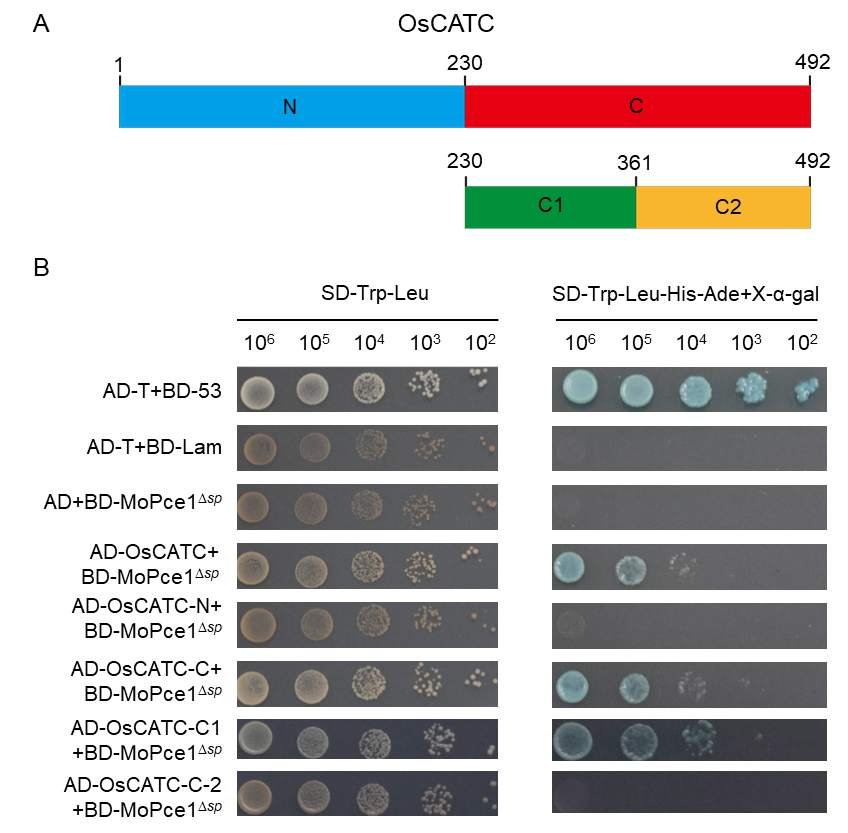


**Figure S5.** Mapping of the fragment required for the interaction between OsCATC and MoPce1. (A) Schematic diagram of truncated OsCATC protein constructs. For mapping of fragment involved in the interaction between OsCATC and MoPce1, the OsCATC was divided into three fragments: N fragment (1 – 230 aa), C1 fragment (231 -360 aa) and C2 fragment (361 – 492 aa). (B) Yeast two-hybrid (Y2H) assay validating the involvement of OsCATC_C1 in the interaction between OsCATC and MoPce1.
