# Supplementary material for: A Core Effector MoPce1 Is Required for the Pathogenicity of Magnaporthe oryzae by Modulating Catalase‐Mediated H2O2 Homeostasis in Rice
Source: Mol Plant Pathol. 2026 Jan 16;27(1):e70206. doi: 10.1111/mpp.70206 (PMC12811410; doi:10.1111/mpp.70206)
Supplement: Supplementary file 8 — Table S3: The colony diameter of ΔMopce1 strain. [file MPP-27-e70206-s015.docx]

Table S3 The colony diameter ofΔ*Mopce1* strain.

| Strain name | Colony diameter (mm) |
| --- | --- |
| Guy11 | 47.5±0.25 |
| *ΔMopce1* | 46.88±0.45 |
| *ΔMopce1-comp* | 47.5±0.13 |
