# Supplementary material for: A Core Effector MoPce1 Is Required for the Pathogenicity of Magnaporthe oryzae by Modulating Catalase‐Mediated H2O2 Homeostasis in Rice
Source: Mol Plant Pathol. 2026 Jan 16;27(1):e70206. doi: 10.1111/mpp.70206 (PMC12811410; doi:10.1111/mpp.70206)
Supplement: Supplementary file 9 — Table S4: The conidiation of ΔMopce1 strain. [file MPP-27-e70206-s005.docx]

Table S4 The conidiation of Δ*Mopce1* strain.

| Strain name | Spore count |
| --- | --- |
| Guy11 | 214.22±6.17 |
| *ΔMopce1* | 204.00±4.16 |
| *ΔMopce1-comp* | 205.33±13.33 |
