# Supplementary material for: A Core Effector MoPce1 Is Required for the Pathogenicity of Magnaporthe oryzae by Modulating Catalase‐Mediated H2O2 Homeostasis in Rice
Source: Mol Plant Pathol. 2026 Jan 16;27(1):e70206. doi: 10.1111/mpp.70206 (PMC12811410; doi:10.1111/mpp.70206)
Supplement: Supplementary file 10 — Table S5: The percentage of lesion types of ΔMopce1 strain. [file MPP-27-e70206-s019.docx]

Table S5 The percentage of lesion types of Δ*Mopce1* strain.

| Strain name | Type1（%） | Type2（%） | Type3（%） | Type4（%） | Type5（%） |
| --- | --- | --- | --- | --- | --- |
| Guy11 | 17.33±6.11 | 30.33±5.86 | 36.67±0.58 | 11.67±3.51 | 4.00±2.00 |
| Δ*Mopce1* | 32.67±7.02*** | 40.00±5.29* | 20.00±4.00**** | 7.00±2.65 | 0.33±0.58 |
| Δ*Mopce1-comp* | 20.67±4.16 | 28.00±3.46 | 31.67±2.89 | 14.33±2.03 | 5.33±3.06 |

Note: Statistical significance was determined using two-way ANOVA followed by Dunnett’s multiple comparisons test (simple effects within columns), comparing ΔMopce1 and ΔMopce1-comp with Guy11.*p <0.05. ***p <0.001, ****p <0.0001
