# Supplementary material for: A Core Effector MoPce1 Is Required for the Pathogenicity of Magnaporthe oryzae by Modulating Catalase‐Mediated H2O2 Homeostasis in Rice
Source: Mol Plant Pathol. 2026 Jan 16;27(1):e70206. doi: 10.1111/mpp.70206 (PMC12811410; doi:10.1111/mpp.70206)
Supplement: Supplementary file 11 — Table S6: The lesion size caused by ΔMopce1 strains ectopically expressed the green fluorescence protein (GFP) fused MoPCE1. [file MPP-27-e70206-s022.docx]

Table S6 The lesion size caused by Δ*Mopce1* strains ectopically expressed the green fluorescence protein (GFP) fused *MoPCE1*.

| Strain name | Lesion area (mm^2)^ |
| --- | --- |
| Guy11 | 182.67±10.79 |
| Δ*Mopce1* | 50.00±2.00**^****^** |
| ΔMopce1/MoPce1-GFP | 157.00±17.35 |
| Δ*Mopce1/GFP-MoPce1* | 148.67±14.05 |

Note:****p <0.0001. Statistical analysis was performed using one-way ANOVA followed by Dunnett’s multiple comparisons test, with Guy11 as the control group
