# Supplementary material for: A Core Effector MoPce1 Is Required for the Pathogenicity of Magnaporthe oryzae by Modulating Catalase‐Mediated H2O2 Homeostasis in Rice
Source: Mol Plant Pathol. 2026 Jan 16;27(1):e70206. doi: 10.1111/mpp.70206 (PMC12811410; doi:10.1111/mpp.70206)
Supplement: Supplementary file 12 — Table S7: The relative biomass of lesions caused by ΔMopce1 strains ectopically expressed the green fluorescence protein (GFP) fused MoPCE1. [file MPP-27-e70206-s006.docx]

Table S7 The relative biomass of lesions caused by Δ*Mopce1* strains ectopically expressed the green fluorescence protein (GFP) fused *MoPCE1*.

| Strain name | Relative biomass |
| --- | --- |
| Guy11 | 18.90±2.11 |
| *ΔMopce1* | 4.30±0.70**^****^** |
| *ΔMopce1/MoPce1-GFP* | 17.21±2.34 |
| *ΔMopce1/GFP-MoPce1* | 16.45±1.04 |

Note: Statistical analysis was performed using one-way ANOVA followed by Dunnett’s multiple comparisons test, with Guy11 as the control group.****p <0.0001
