# Supplementary material for: A Core Effector MoPce1 Is Required for the Pathogenicity of Magnaporthe oryzae by Modulating Catalase‐Mediated H2O2 Homeostasis in Rice
Source: Mol Plant Pathol. 2026 Jan 16;27(1):e70206. doi: 10.1111/mpp.70206 (PMC12811410; doi:10.1111/mpp.70206)
Supplement: Supplementary file 13 — Table S8: The relative expression level of MoPCEΔsp in transgenic plants. [file MPP-27-e70206-s004.docx]

Table S8 The relative expression level of *MoPCE^Δsp^* in transgenic plants.

| Transgenic line | Relative expression level |
| --- | --- |
| *MoPCE1-OX*1 | 37444.10±3263.38**^****^** |
| *MoPCE1-OX*2 | 28246.50±538.93**^****^** |
| *MoPCE1-OX*3 | 24307.56±2106.61**^****^** |
| *MoPCE1-OX*4 | 14520.31±1230.46**^****^** |
| *MoPCE1-OX*5 | 38054.63±1185.59**^****^** |
| *MoPCE1-OX*6 | 17387.76±623.82**^****^** |
| *MoPCE1-OX*7 | 18379.17±742.49**^****^** |
| *MoPCE1-OX*8 | 38496.80±2225.21**^****^** |
| *MoPCE1-OX*9 | 21957.88±999.81**^****^** |
| ZH11 | 1.00±0.00 |

Note: Statistical analysis was performed using one-way ANOVA followed by Dunnett’s multiple comparisons test, with ZH11 as the control group.****p <0.0001
