# Supplementary material for: A Core Effector MoPce1 Is Required for the Pathogenicity of Magnaporthe oryzae by Modulating Catalase‐Mediated H2O2 Homeostasis in Rice
Source: Mol Plant Pathol. 2026 Jan 16;27(1):e70206. doi: 10.1111/mpp.70206 (PMC12811410; doi:10.1111/mpp.70206)
Supplement: Supplementary file 14 — Table S9: The lesion size on MoPCEΔsp‐OX transgenic plants caused by M. oryzae inoculation. [file MPP-27-e70206-s014.docx]

Table S9 The lesion size on *MoPCE^Δsp^-OX* transgenic plants caused by *M. oryzae* inoculation.

| Rice name | Lesion area（mm^2^） |
| --- | --- |
| ZH-11 | 112.44±4.01 |
| *MoPCE1-OX3* | 126.10±4.44 |
| *MoPCE1-OX4* | 169.35±5.01**^**^** |
| *MoPCE1-OX5* | 206.55±6.11 |

Note: Statistical analysis was performed using one-way ANOVA followed by Dunnett’s multiple comparisons test, with ZH11 as the control group.**p <0.01.
