# Supplementary material for: A Core Effector MoPce1 Is Required for the Pathogenicity of Magnaporthe oryzae by Modulating Catalase‐Mediated H2O2 Homeostasis in Rice
Source: Mol Plant Pathol. 2026 Jan 16;27(1):e70206. doi: 10.1111/mpp.70206 (PMC12811410; doi:10.1111/mpp.70206)
Supplement: Supplementary file 15 — Table S10: The relative biomass of lesions on MoPCEΔsp‐OX transgenic plants caused by M. oryzae inoculation. [file MPP-27-e70206-s003.docx]

Table S10 The relative biomass of lesions on *MoPCE^Δsp^-OX* transgenic plants caused by *M. oryzae* inoculation.

| Rice name | Relative biomass |
| --- | --- |
| ZH-11 | 16.72±0.77 |
| *MoPCE1-OX3* | 17.14±0.58 |
| *MoPCE1-OX4* | 18.13±2.42**^*^** |
| *MoPCE1-OX5* | 27.92±1.80**^***^** |

Note: Statistical analysis was performed using one-way ANOVA followed by Dunnett’s multiple comparisons test, with ZH11 as the control group. *p <0.05; ***p <0.001.
