# Supplementary material for: A Core Effector MoPce1 Is Required for the Pathogenicity of Magnaporthe oryzae by Modulating Catalase‐Mediated H2O2 Homeostasis in Rice
Source: Mol Plant Pathol. 2026 Jan 16;27(1):e70206. doi: 10.1111/mpp.70206 (PMC12811410; doi:10.1111/mpp.70206)
Supplement: Supplementary file 16 — Table S11: The luminescence generated from the wild type and MoPCEΔsp‐OX transgenic plants in response to flg22. [file MPP-27-e70206-s018.docx]

Table S11 The luminescence generated from the wild type and *MoPCE^Δsp^-OX* transgenic plants in response to flg22.

| ZH11-Water | *MoPCE1-OX*-Water | ZH11-flg22 | *MoPCE1-OX*-flg22 |
| --- | --- | --- | --- |
| 7209.95±206.22 | 246.06±38.72**^*^** | 9750.27±437.64 | 11555.06±1906.85 |
| 8047.13±24.34 | 169.42±2.77**^*^** | 10730.90±151.60 | 12053.60±1407.00 |
| 8320.80±303.75 | 201.93±10.13**^**^** | 19788.00±1743.20**^****^** | 14703.45±812.75**^*^** |
| 8953.75±364.61 | 239.10±9.72**^**^** | 39836.80±3544.10**^****^** | 21052.95±1666.05**^****^** |
| 9416.65±1095.76 | 220.47±45.75**^**^** | 54924.60±970.40**^****^** | 29036.30±3832.40**^****^** |
| 9612.39±524.12 | 259.04±8.41**^**^** | 60709.20±2294.20**^****^** | 34907.10±3890.60**^****^** |
| 10074.68±788.92 | 347.08±0.56**^**^** | 65950.10±3641.40**^****^** | 39445.00±4397.20**^****^** |
| 11128.15±764.75 | 345.30±22.03**^***^** | 68309.35±4932.05**^****^** | 42236.00±3804.4**^****^** |
| 11612.25±1458.85 | 395.00±60.25**^***^** | 69698.00±6333.70**^****^** | 43682.90±2879.50**^****^** |
| 12365.40±896.50 | 321.93±40.61**^****^** | 67453.40±8990.80**^****^** | 43277.05±2556.75**^****^** |
| 12490.10±788.50 | 259.17±58.14**^****^** | 63276.95±6724.35**^****^** | 43887.35±2735.75**^****^** |
| 12652.00±1065.10 | 218.55±41.06**^****^** | 60552.40±7926.6**^****^** | 42509.50±2361.50**^****^** |
| 13372.15±1126.15 | 349.06±29.08**^****^** | 55238.95±7413.75**^****^** | 40132.15±1679.55**^****^** |
| 12765.70±1697.10 | 374.95±37.29**^****^** | 52085.15±6731.75**^****^** | 38152.30±1352.70**^****^** |
| 13566.70±1715.60 | 423.19±53.93**^****^** | 48261.25±5978.25**^****^** | 35821.25±411.55**^****^** |
| 13804.65±743.85 | 453.69±42.36**^****^** | 44902.00±7464.70**^****^** | 35188.90±1056.60**^****^** |
| 14031.90±500.30 | 342.80±33.32**^****^** | 42173.60±6281.30**^****^** | 34128.00±688.40**^****^** |
| 13898.80±622.10 | 412.40±69.61**^****^** | 39963.70±5493.70**^****^** | 32680.25±423.35**^****^** |
| 14520.95±1014.95 | 426.28±96.27**^****^** | 38745.90±6007.50**^****^** | 32034.35±285.25**^****^** |
| 14475.35±1392.65 | 421.98±128.11**^****^** | 38042.25±4926.15**^****^** | 30939.80±1.20**^****^** |
| 14626.50±1316.60 | 467.27±56.44**^****^** | 36485.80±7129.20**^****^** | 30994.45±19.45**^****^** |
| 14506.75±1228.75 | 523.17±69.80**^****^** | 37875.05±4917.75**^****^** | 30020.00±335.90**^****^** |
| 14647.25±1476.15 | 568.33±53.92**^****^** | 38525.15±5493.15**^****^** | 29482.90±147.70**^****^** |
| 15027.20±1022.60 | 481.61±87.48**^****^** | 40350.95±5880.65**^****^** | 30239.20±205.60**^****^** |
| 15263.40±1350.30 | 569.67±33.21**^****^** | 40502.90±5600.20**^****^** | 29636.05±1108.95**^****^** |
| 15415.90±994.10 | 612.69±135.66**^****^** | 39410.40±7700.20**^****^** | 30002.55±71.05**^****^** |
| 15643.75±1006.35 | 619.91±20.37**^****^** | 40774.60±6002.90**^****^** | 29977.80±921.40**^****^** |
| 15444.20±742.30 | 565.22±109.62**^****^** | 41210.60±6604.20**^****^** | 30959.70±1342.00**^****^** |
| 15843.95±1483.25 | 561.79±39.23**^****^** | 41879.40±5853.70**^****^** | 31230.30±1014.70**^****^** |
| 15082.60±22.50 | 635.47±38.33**^****^** | 42129.70±6647.90**^****^** | 32793.10±1985.10**^****^** |
| 15107.80±726.80 | 627.49±20.55**^****^** | 40946.65±6428.25**^****^** | 32175.00±1201.70**^****^** |
| 15271.95±695.15 | 668.05±9.88**^****^** | 42136.90±7170.20**^****^** | 32804.05±918.05**^****^** |
| 15547.25±805.35 | 692.86±84.90**^****^** | 41586.65±7357.85**^****^** | 34174.00±1434.50**^****^** |
| 15207.40±595.10 | 745.22±25.57**^****^** | 41100.30±6472.30**^****^** | 34197.80±1964.30**^****^** |
| 15753.50±696.90 | 654.28±65.03**^****^** | 41196.10±7513.40**^****^** | 34674.10±1302.90**^****^** |

Note: Statistical significance was assessed using two-way ANOVA followed by Dunnett’s multiple comparisons test (simple effects within rows), with ZH11-Water as the control. *p<0.05; **p<0.01; ***p<0.001; ****p<0.0001.
