# Supplementary material for: A Core Effector MoPce1 Is Required for the Pathogenicity of Magnaporthe oryzae by Modulating Catalase‐Mediated H2O2 Homeostasis in Rice
Source: Mol Plant Pathol. 2026 Jan 16;27(1):e70206. doi: 10.1111/mpp.70206 (PMC12811410; doi:10.1111/mpp.70206)
Supplement: Supplementary file 17 — Table S12: The luminescence generated from the wild type and MoPCEΔsp‐OX transgenic plants in response to Chitin. [file MPP-27-e70206-s024.docx]

Table S12 The luminescence generated from the wild type and *MoPCE^Δsp^-OX* transgenic plants in response to Chitin.

| ZH11-Water | *MoPCE1-OX*-Water | ZH11-Chitin | *MoPCE1-OX*-Chitin |
| --- | --- | --- | --- |
| 6142.83±135.04 | 1752.94±335.89 | 7777.04±802.47 | 6884.08±1123.86 |
| 7990.05±140.86 | 2110.08±314.94**^*^** | 9010.09±998.32 | 9753.43±919.68 |
| 8328.57±343.31 | 2516.72±425.77**^*^** | 13179.80±1547.10 | 14802.45±1263.95**^**^** |
| 8515.40±81.75 | 2603.28±277.87**^*^** | 18634.70±1930.60**^****^** | 18696.90±1858.30**^****^** |
| 8982.77±563.37 | 2716.21±194.73**^*^** | 23580.00±1213.40**^****^** | 22770.30±3456.20**^****^** |
| 9246.46±198.45 | 3162.46±439.97**^*^** | 29065.90±757.40**^****^** | 25499.15±3387.75**^****^** |
| 10028.28±94.62 | 3355.73±56.21**^**^** | 32273.05±916.15**^****^** | 27854.70±4788.40**^****^** |
| 10215.57±670.64 | 3444.12±28.79**^**^** | 35684.25±1220.45**^****^** | 28957.05±4983.95**^****^** |
| 10485.10±149.40 | 3728.02±150.05**^**^** | 35741.90±119.00**^****^** | 29693.95±5237.45**^****^** |
| 10964.25±168.05 | 4076.34±161.75**^**^** | 37048.70±15.80**^****^** | 30056.05±5685.55**^****^** |
| 10365.45±208.05 | 4478.59±226.34**^*^** | 36870.60±775.80**^****^** | 29091.50±5332.90**^****^** |
| 11390.10±538.80 | 4572.47±202.49**^**^** | 37056.80±312.40**^****^** | 29154.00±5926.90**^****^** |
| 11629.55±398.95 | 4550.95±214.95**^**^** | 36244.10±463.70**^****^** | 27402.10±6365.30**^****^** |
| 12164.60±122.90 | 4715.37±41.13**^**^** | 35572.15±646.25**^****^** | 26765.60±6123.80**^****^** |
| 12254.10±400.70 | 5211.70±234.13**^**^** | 35067.20±293.70**^****^** | 25708.15±5536.25**^****^** |
| 12340.05±315.35 | 5342.24±446.63**^**^** | 33853.75±323.55**^****^** | 24445.50±5656.10**^****^** |
| 13015.60±271.20 | 5734.36±25.21**^**^** | 32398.10±35.20**^****^** | 23210.60±6547.60**^****^** |
| 12605.45±695.15 | 6132.01±85.26**^**^** | 31748.80±1469.70**^****^** | 23040.35±6178.95**^****^** |
| 11812.05±597.15 | 6030.64±28.07**^*^** | 31607.55±1142.85**^****^** | 21516.45±6616.35**^****^** |
| 11280.25±1263.15 | 6536.77±429.58 | 30835.30±926.80**^****^** | 21127.15±6125.05**^****^** |
| 11382.25±1476.56 | 6849.11±189.91 | 31101±460.10**^****^** | 20116.15±6600.65**^***^** |
| 11355.71±1442.10 | 7630.82±188.78 | 29589.85±1194.45**^****^** | 19759.85±5465.15**^***^** |
| 10893.97±1310.83 | 7462.51±110.95 | 28870.55±872.65**^****^** | 18812.05±5762.75**^***^** |
| 10865.40±1940.60 | 7789.86±62.69 | 28419.10±525.60**^****^** | 18377.85±4928.25**^**^** |
| 11113.20±2618.50 | 8075.34±51.73 | 26806.00±1008.10**^****^** | 17666.60±5174.80**^**^** |
| 10734.07±2469.13 | 7941.80±184.72 | 26725.15±904.95**^****^** | 16950.70±5023.70**^*^** |
| 11302.80±2246.31 | 8558.45±799.82 | 25503.55±56.85**^****^** | 16781.00±5014.70**^*^** |
| 11296.57±2477.23 | 9027.35±571.95 | 24783.55±1217.75**^****^** | 17055.60±5321.70**^*^** |
| 11209.08±2232.63 | 8983.84±748.50 | 24633.75±515.15**^****^** | 15694.45±3905.65 |
| 11140.68±2037.12 | 8765.54±231.38 | 23976.90±740.80**^****^** | 16127.25±4852.85 |
| 10987.32±2143.38 | 8747.43±234.62 | 22463.75±548.95**^****^** | 15849.85±4351.25 |
| 11587.86±2679.94 | 9657.87±764.44 | 22980.00±953.00**^****^** | 15479.25±4543.75 |
| 11726.13±2557.47 | 9114.86±1101.04 | 22949.80±1106.20**^****^** | 15130.80±3951.30 |
| 12211.57±2719.53 | 9692.99±849.31 | 21070.45±1074.75**^***^** | 14681.55±3887.55 |
| 12449.83±2490.18 | 9420.63±630.08 | 20517.80±94.90**^***^** | 15147.80±4186.00 |

Note: Statistical significance was assessed using two-way ANOVA followed by Dunnett’s multiple comparisons test (simple effects within rows), with ZH11-Water as the control. *p<0.05; **p<0.01; ***p<0.001; ****p<0.0001.
