# Supplementary material for: A Core Effector MoPce1 Is Required for the Pathogenicity of Magnaporthe oryzae by Modulating Catalase‐Mediated H2O2 Homeostasis in Rice
Source: Mol Plant Pathol. 2026 Jan 16;27(1):e70206. doi: 10.1111/mpp.70206 (PMC12811410; doi:10.1111/mpp.70206)
Supplement: Supplementary file 18 — Table S13: The relative expression level of PR genes in the MoPCEΔsp‐OX transgenic plants challenged with or without M. oryzae . [file MPP-27-e70206-s010.docx]

Table S13 The relative expression level of PR genes in the *MoPCE^Δsp^-OX* transgenic plants challenged with or without *M. oryzae*.

|  | Relative expression | | | |
| --- | --- | --- | --- | --- |
|  | *PR2* | *NH1* | *CEBIP* | *BIMK2* |
| H_2_0 0h ZH11 | 1±0.00 | 1±0.00 | 1±0.00 | 1±0.00 |
| H_2_0 0h *MoPCE1^Δsp^-OX* | 1.29±0.03 | 1.07±0.22 | 1.04±0.06 | 0.97±0.17 |
| H_2_0 48h ZH11 | 10.20±0.70 | 7.61±2.78 | 0.76±0.17 | 4.93±0.29 |
| H_2_0 48h *MoPCE1^Δsp^-OX* | 11.03±1.54 | 5.93±1.21 | 0.81±0.09 | 4.99±1.18 |
| Guy11 48h ZH11 | 81.47±4.70^****^ | 30.31±6.91^****^ | 0.99±0.22 | 50.72±8.34^****^ |
| Guy11 48h *MoPCE1^Δsp^-OX* | 37.63±6.24^****^ | 13.07±1.72 | 0.78±0.09 | 10.86±2.00 |

Note: Statistical analysis was performed using one-way ANOVA followed by Dunnett’s multiple comparisons test, with H₂O 0 h ZH11 as the control group.**p<0.01; ***p<0.001; ****p<0.0001.
