# Supplementary material for: A Core Effector MoPce1 Is Required for the Pathogenicity of Magnaporthe oryzae by Modulating Catalase‐Mediated H2O2 Homeostasis in Rice
Source: Mol Plant Pathol. 2026 Jan 16;27(1):e70206. doi: 10.1111/mpp.70206 (PMC12811410; doi:10.1111/mpp.70206)
Supplement: Supplementary file 19 — Table S14: The list of putative MoPce1 interacting proteins screened through Yeast‐two‐hybrid. [file MPP-27-e70206-s017.docx]

Table S14 The list of putative MoPce1 interacting proteins screened through CoIP/MS.

| Code | Accession Number | Gene Product | Number of unique peptides |
| --- | --- | --- | --- |
| PIP1 | Os07g0152900 | peroxisomal (S)-2-hydroxy-acid oxidase GLO5-like | 16 |
| PIP2 | Os12g0274700 | ribulose bisphosphate carboxylase small chain A, chloroplastic-like | 14 |
| PIP3 | Os03g0786100 | peroxisomal (S)-2-hydroxy-acid oxidase GLO1-like | 13 |
| PIP4 | Os03g0276500 | heat shock cognate 70 kDa protein 2 | 9 |
| PIP5 | Os07g0301200 | DEAD-box ATP-dependent RNA helicase 5-like | 15 |
| PIP6 | Os03g0802650 | DEAD-box ATP-dependent RNA helicase 27-like | 13 |
| PIP7 | Os08g0154600 | DNA topoisomerase 1 beta | 6 |
| PIP8 | Os03g0131200 | catalase-1 | 18 |
| PIP9 | Os02g0553200 | probable L-ascorbate peroxidase 8, chloroplastic | 13 |
| PIP10 | Os08g0549100 | probable L-ascorbate peroxidase 4, peroxisomal | 7 |
| PIP11 | [Os03g0851700](https://rapdb.dna.affrc.go.jp/locus/?name=Os03g0851700) | ankyrin repeat domain containing protein, expressed | 4 |
| PIP12 | [Os01g0948900](https://rapdb.dna.affrc.go.jp/locus/?name=Os01g0948900) | BTBA3 - Bric-a-Brac,Tramtrack, Broad Complex BTB domain with Ankyrin repeat region, expressed | 1 |
| PIP13 | Os01g0172400 | phospholipase D alpha 1-like | 26 |
| PIP14 | Os06g0604400 | phospholipase D alpha 2-like | 11 |
| PIP15 | Os07g0694000 | phosphoinositide phospholipase C 2 | 9 |
| PIP16 | Os01g0895600 | calreticulin-3 | 13 |
| PIP17 | Os04g0402100 | calnexin homolog | 10 |
| PIP18 | Os05g0507300 | calreticulin-3 | 7 |

Note：The number of unique peptides represents the average value calculated from three independent samples.
