# Supplementary material for: A Core Effector MoPce1 Is Required for the Pathogenicity of Magnaporthe oryzae by Modulating Catalase‐Mediated H2O2 Homeostasis in Rice
Source: Mol Plant Pathol. 2026 Jan 16;27(1):e70206. doi: 10.1111/mpp.70206 (PMC12811410; doi:10.1111/mpp.70206)
Supplement: Supplementary file 20 — Table S15: The lesion size on oscatc transgenic plants caused by M. oryzae inoculation. [file MPP-27-e70206-s007.docx]

Table S15 The lesion size on *oscatc* transgenic plants caused by *M. oryzae* inoculation.

| Rice name | Lesion area（mm^2^） |
| --- | --- |
| ZH-11 | 84.55±4.18 |
| *Oscatc*-7 | 48.39±3.81^***^ |
| *Oscatc*-10 | 45.95±2.51^***^ |

Note: Statistical analysis was performed using one-way ANOVA followed by Dunnett’s multiple comparisons test, with ZH11 as the control group. ***p<0.001.
