# Supplementary material for: A Core Effector MoPce1 Is Required for the Pathogenicity of Magnaporthe oryzae by Modulating Catalase‐Mediated H2O2 Homeostasis in Rice
Source: Mol Plant Pathol. 2026 Jan 16;27(1):e70206. doi: 10.1111/mpp.70206 (PMC12811410; doi:10.1111/mpp.70206)
Supplement: Supplementary file 21 — Table S16: The relative biomass of lesions on oscatc transgenic plants caused by M. oryzae inoculation. [file MPP-27-e70206-s009.docx]

Table S16 The relative biomass of lesions on *oscatc* transgenic plants caused by *M. oryzae* inoculation.

| Rice name | Relative biomass |
| --- | --- |
| ZH-11 | 14.15±0.50 |
| *Oscatc*-7 | 4.21±0.34^****^ |
| *Oscatc*-10 | 5.99±0.10^****^ |

Note: Statistical analysis was performed using one-way ANOVA followed by Dunnett’s multiple comparisons test, with ZH11 as the control group. ****p<0.0001
