# Supplementary material for: A Core Effector MoPce1 Is Required for the Pathogenicity of Magnaporthe oryzae by Modulating Catalase‐Mediated H2O2 Homeostasis in Rice
Source: Mol Plant Pathol. 2026 Jan 16;27(1):e70206. doi: 10.1111/mpp.70206 (PMC12811410; doi:10.1111/mpp.70206)
Supplement: Supplementary file 22 — Table S17: The luminescence generated from the wild type and oscatc plants in response to flg22. [file MPP-27-e70206-s016.docx]

Table S17 The luminescence generated from the wild type and *oscatc* plants in response to flg22.

| ZH11-Water | *oscatc*-Water | ZH11-flg22 | *oscatc*-flg22 |
| --- | --- | --- | --- |
| 230.73±75.67 | 517.14±37.13 | 2339.28±1310.81 | 7127.6±5344.36 |
| 332.67±116.29 | 594.36±202 | 2304.52±1392.43 | 7863.41±5825.24 |
| 279.71±165.89 | 625.85±67.07 | 2750.48±1703.59 | 8713.61±6779.43 |
| 344.9±73.16 | 560.31±204.79 | 2705.44±1472.4 | 9937.43±7667.86 |
| 296.52±66.76 | 643.52±73.69 | 2880.64±1579.18 | 14758.92±13830.44 |
| 331.72±227.2 | 755.73±97.77 | 3841.29±2358.95 | 52232.17±53216.01 |
| 443.11±187.86 | 995.09±270.16 | 10276.26±7170.66 | 181955.4±130702.59^****^ |
| 475.24±217.06 | 1075.06±260.99 | 29297.8±16674.96 | 411311.67±164467.92^****^ |
| 446.67±233.61 | 1312.06±211.65 | 54952.43±24225.93 | 606865±113510.29^****^ |
| 634.51±247.24 | 1416.85±99.31 | 79997.37±30028.85^*^ | 717623±74626.58^****^ |
| 663.99±401.76 | 1556.31±56.66 | 102162.87±33454.72^**^ | 759944.67±53477.68^****^ |
| 749.06±413.88 | 1441.04±22.85 | 116495.1±34895.67^**^ | 869878.33±54819.12^****^ |
| 704.06±314.87 | 1588.13±274.06 | 132186.83±32441.53^***^ | 896054.33±57590.22^****^ |
| 738.71±259.53 | 1583.66±134.34 | 141346±31964.22^***^ | 891284.33±37036.91^****^ |
| 819.73±421.34 | 1591.23±245.9 | 146049.33±34361.54^****^ | 870940.33±52229.41^****^ |
| 729.85±451.9 | 1480.02±127.37 | 148702±29163.67^****^ | 788722±52847.2^****^ |
| 807.36±548.86 | 1590.65±245.95 | 145626±27069.26^****^ | 789599.67±67879.74^****^ |
| 769.48±424.2 | 1465.77±67.3 | 140812.67±27385.8^***^ | 760683±72691.31^****^ |
| 730.34±358.82 | 1364.3±238.46 | 132393.33±22830.73^***^ | 720390.33±71065.96^****^ |
| 763.05±462.85 | 1587.05±155.76 | 124902±22131.72^***^ | 684399.67±76006.94^****^ |
| 750.52±395.2 | 1321.5±313.01 | 114574.33±21521.42^**^ | 622248.67±52667.34^****^ |
| 777.66±489.49 | 1400.4±216.94 | 107523.2±20083.07^**^ | 658277±99607.25^****^ |
| 609.2±380.09 | 1460.76±106.4 | 103783.17±18395.6^**^ | 664542±115431.28^****^ |
| 732.85±351.36 | 1126.07±99.49 | 99713.47±17385.79^**^ | 647230.67±128153.52^****^ |
| 659.58±263.94 | 1255.63±196.79 | 99275.3±17823.24^**^ | 634531.33±115824.18^****^ |
| 718.57±358.63 | 1234.37±352.59 | 97716.4±17886.48^*^ | 561599±69774.36^****^ |
| 659.49±434.12 | 1320.12±136.83 | 95143.23±16106.43^*^ | 560743.67±90494.56^****^ |
| 702.83±526.81 | 1154.24±139.6 | 96547.37±17872.33^*^ | 516230.67±78258.17^****^ |
| 710.29±387.15 | 1179.43±263.06 | 93613.63±14318.51^*^ | 471208.33±78208.08^****^ |
| 760.79±451.46 | 1148.07±62.48 | 90059.1±14729.46^*^ | 425136.67±74117.22^****^ |
| 567.67±307.04 | 1059.14±342.35 | 87605.8±14751.93^*^ | 367774.67±61981.78^****^ |
| 579.43±330.62 | 1004.47±88.12 | 81801.77±12872.15^*^ | 339828±82462.63^****^ |
| 663.81±247.71 | 1156.24±114.22 | 77482.3±11415.46 | 302282.33±79600.05^****^ |
| 568.71±295 | 1055.25±300.2 | 71252.87±12081.7 | 264777.33±81871.31^****^ |
| 490.24±275.26 | 890.61±76.44 | 68600.17±10031.8 | 234795.67±80934.24^****^ |

Note: Statistical significance was assessed using two-way ANOVA followed by Dunnett’s multiple comparisons test (simple effects within rows), with ZH11-Water as the control. *p<0.05; **p<0.01; ***p<0.001; ****p<0.0001.
