# Supplementary material for: A Core Effector MoPce1 Is Required for the Pathogenicity of Magnaporthe oryzae by Modulating Catalase‐Mediated H2O2 Homeostasis in Rice
Source: Mol Plant Pathol. 2026 Jan 16;27(1):e70206. doi: 10.1111/mpp.70206 (PMC12811410; doi:10.1111/mpp.70206)
Supplement: Supplementary file 23 — Table S18: The luminescence generated from the wild type and oscatc plants in response to Chitin. [file MPP-27-e70206-s020.docx]

Table S18 The luminescence generated from the wild type and *oscatc* plants in response to Chitin.

| ZH11-Water | *oscatc*-Water | ZH11-Chitin | *oscatc*-Chitin |
| --- | --- | --- | --- |
| 155.82±46.44 | 499.14±276.67 | 3399.31±3370.71 | 14408.51±6018.71 |
| 153.65±85.77 | 348.13±103.37 | 3335.05±2996 | 13231.12±5680.41 |
| 173.94±83.01 | 335.05±124.48 | 2859.41±2161.32 | 13094.87±5645.34 |
| 233.6±57.94 | 397.67±199.17 | 5955.85±3678.17 | 14138.48±6529.52 |
| 185.24±89.5 | 322.05±180.01 | 27302.67±13484.85 | 33118.53±21074.01 |
| 251.47±102.42 | 375.22±66.61 | 59568.8±13441.38 | 102198.73±67652.35^**^ |
| 181.96±49.8 | 501.81±217.09 | 87895.9±5926.43^**^ | 230025±123278.16^****^ |
| 268.11±194.12 | 544.56±91.03 | 105889.07±7316.88^**^ | 389336.67±149184.79^****^ |
| 318.46±169.96 | 647.27±53.13 | 115755.33±9534.12^***^ | 535162.67±149103.25^****^ |
| 301.68±110.94 | 817.32±111.47 | 117178.67±11122.77^***^ | 652615±144314.64^****^ |
| 302.62±147.51 | 806.02±53.58 | 119468±14312.4^***^ | 653348.33±118614.03^****^ |
| 359.11±182.88 | 959.43±80.58 | 117526.5±18697.48^***^ | 745100±109060.61^****^ |
| 390.26±221.06 | 990.55±141.41 | 107630.7±17254.05^**^ | 710471.33±88597.72^****^ |
| 341.39±129.63 | 1001.76±164.11 | 95930.9±17419.71^**^ | 641033.33±82793.2^****^ |
| 370.94±192.57 | 1169.78±153.04 | 83888.4±14779.93^*^ | 574727±73205.65^****^ |
| 355.84±198.24 | 1271.53±296.93 | 73027.53±14569.75^*^ | 464868±62212.37^****^ |
| 430.41±157.33 | 1203.86±158.25 | 63002.77±9976.94 | 462435.67±69349.48^****^ |
| 430.08±169.52 | 1268.2±98.77 | 55896.83±7977.99 | 418204.67±68194.32^****^ |
| 385.45±176.06 | 1343.17±240.04 | 50395±6442.72 | 380269±63610.6^****^ |
| 463.08±146.88 | 1414.03±120.53 | 45549.4±6891.71 | 358336.33±65427.96^****^ |
| 374.64±187.78 | 1552.73±415.69 | 41235.7±5476.39 | 319079.33±59742.68^****^ |
| 315.75±59.69 | 1408.75±278.11 | 36972.63±4621.26 | 317160.33±57785.59^****^ |
| 299.28±123.32 | 1360.69±426.13 | 34072.9±4349.43 | 298516.33±54016.77^****^ |
| 335.76±192.06 | 1331.11±438.91 | 32526.83±3574.16 | 279725.67±53340.47^****^ |
| 321.43±181.74 | 1326.51±376.37 | 29659.77±3787.83 | 265656.67±50101.59^****^ |
| 345.05±196.25 | 1536.33±675.8 | 27795.13±3085.88 | 236165.33±46700.47^****^ |
| 257.36±22.23 | 1289.62±349.63 | 25748.67±3309.32 | 234437±44564.13^****^ |
| 329.26±109.28 | 1328.29±492.35 | 24189.27±3108.2 | 223443±43918.46^****^ |
| 360.95±198.13 | 1227.01±345.32 | 22468.27±3124.37 | 207680.33±36138.08^****^ |
| 291.11±48.5 | 1401.12±293.94 | 20908.5±3325.51 | 199202.33±37519.29^****^ |
| 257.94±143.36 | 1347.91±626.66 | 19743±3613.79 | 182049.33±38021.65^****^ |
| 332.65±73.41 | 1282.86±360.41 | 18472.07±2866.68 | 178900.67±32183.66^****^ |
| 316.77±51.41 | 1142.24±356.35 | 17347.5±3278.12 | 174182.67±35673.59^****^ |
| 267.48±71.66 | 1224±131.96 | 16540.27±2932.65 | 164125.33±35159.75^****^ |
| 248.09±149.59 | 1249.66±267.96 | 15086.27±2585.54 | 157913±35164.86^****^ |

Note: Statistical significance was assessed using two-way ANOVA followed by Dunnett’s multiple comparisons test (simple effects within rows), with ZH11-Water as the control. *p<0.05; **p<0.01; ***p<0.001; ****p<0.0001.
